# Right Posterior Temporal Cortex Supports Integration of Phonetic and Talker Information

**DOI:** 10.1162/nol_a_00091

**Published:** 2023-03-08

**Authors:** Sahil Luthra, James S. Magnuson, Emily B. Myers

**Affiliations:** Department of Psychological Sciences, University of Connecticut, Storrs, CT, USA; Basque Center on Cognition Brain and Language (BCBL), Donostia-San Sebastián, Spain; Ikerbasque, Basque Foundation for Science, Bilbao, Spain; Speech, Language, and Hearing Sciences, University of Connecticut, Storrs, CT, USA

**Keywords:** talker information, phonetic processing, speech perception, perceptual learning, right hemisphere, fMRI

## Abstract

Though the right hemisphere has been implicated in talker processing, it is thought to play a minimal role in phonetic processing, at least relative to the left hemisphere. Recent evidence suggests that the right posterior temporal cortex may support learning of phonetic variation associated with a specific talker. In the current study, listeners heard a male talker and a female talker, one of whom produced an ambiguous fricative in /s/-biased lexical contexts (e.g., *epi?ode*) and one who produced it in /∫/-biased contexts (e.g., *friend?ip*). Listeners in a behavioral experiment (Experiment 1) showed evidence of lexically guided perceptual learning, categorizing ambiguous fricatives in line with their previous experience. Listeners in an fMRI experiment (Experiment 2) showed differential phonetic categorization as a function of talker, allowing for an investigation of the neural basis of talker-specific phonetic processing, though they did not exhibit perceptual learning (likely due to characteristics of our in-scanner headphones). Searchlight analyses revealed that the patterns of activation in the right superior temporal sulcus (STS) contained information about who was talking and what phoneme they produced. We take this as evidence that talker information and phonetic information are integrated in the right STS. Functional connectivity analyses suggested that the process of conditioning phonetic identity on talker information depends on the coordinated activity of a left-lateralized phonetic processing system and a right-lateralized talker processing system. Overall, these results clarify the mechanisms through which the right hemisphere supports talker-specific phonetic processing.

## INTRODUCTION

Speech scientists have long appreciated that individual talkers can differ considerably in how they produce their speech sounds, such that the acoustics that signal an /ɛ/ (the vowel in “bed”) for one talker might correspond to an /æ/ (as in “bad”) for another (Hillenbrand et al., 1995; Joos, 1948; Peterson & Barney, 1952). Listeners are sensitive to the acoustic-phonetic variability across talkers (Allen & Miller, 2004; Newman et al., 2001; Theodore & Miller, 2010) but nevertheless are able to accurately recognize words spoken by a wide range of talkers (e.g., Liberman et al., 1967). Thus, there must be some mechanism by which listeners accommodate talker variability—that is, some way in which they can condition their interpretation of a speech sound on their knowledge of who is talking. Bayesian accounts posit that listeners accommodate talker variability by maintaining distinct sets of beliefs—that is, a distinct generative model—for how a given talker produces speech sounds; a model may describe an individual talker or a group of talkers that share socio-indexical traits such as age, gender, sexual orientation, and/or national origin (Kleinschmidt, 2019).

High-level information, such as word-level knowledge, plays a critical role in helping listeners adapt to the idiosyncratic ways that different talkers produce speech (Maye et al., 2008; Norris et al., 2003). In a landmark study, Norris et al. (2003) exposed Dutch listeners to a talker who often produced a speech sound that was ambiguous between /s/ and /f/ (denoted /?/). Crucially, one group of listeners only heard the ambiguous phoneme in contexts where lexical information biased their interpretation of the phoneme toward /s/ (contexts like [ra:dɛi?], where [ra:dɛis] (*radijs*) is the Dutch word for radish, but [ra:dɛif] (*radijf) is a nonword), and the other group only heard the ambiguous phoneme in /f/-biased contexts. During an initial exposure phase, listeners were asked to indicate whether these items were real words or nonwords, and they consistently endorsed items with ambiguous phonemes as being real words, suggesting that they used lexical knowledge to guide their immediate interpretation of the ambiguous phoneme (an effect previously shown by Ganong, 1980). After the initial exposure phase, Norris et al. had participants complete a phonetic categorization task with stimuli from an /f/-/s/ nonword–nonword continuum produced by the same talker. They found that participants who had previously heard ambiguous fricatives in /s/-biased contexts were likely to identify similar ambiguous fricatives as /s/, and those who had heard ambiguous fricatives in /f/-biased contexts were more likely to interpret ambiguous tokens as /f/. That is, contexts encountered during exposure allowed listeners to update their beliefs for how that talker produced speech sounds. Work on lexically guided perceptual learning has shown that listeners can simultaneously track the phonetic idiosyncrasies of two different talkers (e.g., if one produces an ambiguous fricative in place of /s/ while another produces an ambiguous fricative in place of /∫/ [“sh”]; Kraljic & Samuel, 2005, 2007; Luthra et al., 2021), consistent with the proposal that listeners can maintain separate generative models for different sets of talkers.

Some insight into the neural mechanisms through which listeners contact generative models to guide speech perception comes from a lexically guided perceptual learning study conducted by Myers and Mesite (2014). The authors found that during phonetic categorization, two regions exhibited differential responses to the ambiguous tokens as a function of previous exposure—the right middle frontal gyrus (MFG) and the right middle temporal gyrus (MTG). The involvement of the right hemisphere may be surprising, given that phonetic processing is primarily supported by regions in the left hemisphere (Rauschecker & Scott, 2009; Turkeltaub & Branch Coslett, 2010). However, prominent models of speech perception, such as the dual stream model (Hickok & Poeppel, 2000, 2004, 2007) point out that at low levels of the language hierarchy (phonology), information may be fairly bilaterally represented, and right temporal regions are often recruited in functional neuroimaging studies of speech perception (Belin et al., 1999; Blumstein et al., 2005; Davis et al., 2011; Giraud et al., 2004; Zatorre et al., 1996). Furthermore, vocal identity processing relies principally on the contributions of right temporal cortex (Belin et al., 2000; Belin & Zatorre, 2003; Schall et al., 2014; von Kriegstein et al., 2003; von Kriegstein & Giraud, 2004) and sometimes places demands on right frontal cortex as well (Andics et al., 2013; Stevens, 2004). Myers and Mesite therefore suggested that the involvement of the right hemisphere in their study might reflect the fact that during the phonetic categorization runs, listeners had to access their beliefs about the idiosyncratic way that this particular talker produced speech sounds.

In a follow-up study, Luthra et al. (2020) performed multivoxel pattern analyses (MVPA) on the data from the lexically guided perceptual learning study by Myers and Mesite (2014) described above. In particular, Luthra et al. trained a machine learning algorithm on the correspondences between phonetic identity and patterns of functional activation for unambiguous stimuli (i.e., clear productions of /asi/ and /a∫i/ that participants heard during the phonetic categorization runs). When the classifier was tested on the patterns of functional activation for the ambiguous stimuli (i.e., those in the middle of the /asi/-/a∫i/ continuum), with trials labeled based on whether the listener had reported /s/ or /∫/ on that particular trial, the classifier achieved above-chance accuracy. That is, the pattern of activation for an ambiguous trial resembled the canonical pattern for whichever endpoint (/s/ or /∫/) the subject reported having heard. Their initial analysis considered a broad set of regions involved in language processing, but exploratory region-of-interest (ROI) analyses found that above-chance accuracy was still observed when the classifier only received information about the activity of left parietal cortex or information about right temporal cortex. These findings provide further evidence for a right hemisphere role—right temporal cortex in particular—in representing a listener’s perceptual interpretation of speech from a talker with systematically atypical productions. Notably, however, listeners in the Myers and Mesite study only heard speech from one talker, so it is unclear whether the involvement of the right temporal cortex is a consequence of listeners contacting a *talker-specific* generative model.

The proposal that phonetic information and talker detail might be integrated in the right temporal cortex is consistent with a growing body of evidence that the right temporal cortex simultaneously represents phonetic detail and talker information (Evans & Davis, 2015; Formisano et al., 2008; von Kriegstein et al., 2010). In a seminal study, Formisano et al. (2008) presented listeners with three different vowels produced by three different talkers while measuring brain responses using fMRI. Using a machine learning technique, they found that the most informative voxels for phonetic classification spanned bilateral temporal areas including the superior temporal gyrus (STG), superior temporal sulcus (STS), and MTG. In contrast, the most informative voxels for talker identity were right-lateralized, located primarily in the right STS, though a small number of voxels in left STS also contributed meaningfully. Crucially, there was a small subset of voxels in which activation patterns were useful in both the classification of phonetic identity *and* of talker identity, with these voxels primarily located in the right STS, though some degree of overlap in the left STS was also observed. The authors emphasized the finding that most voxels that were informative for phonetic classification were not informative for talker classification (and vice versa), suggesting that phonetic information and talker information are segregated relatively early in processing.

Additional evidence for this view comes from a functional MRI study by Evans and Davis (2015). To investigate how different dimensions of the speech signal are encoded across the brain, the authors used representational similarity analyses (Kriegeskorte et al., 2008) to examine the similarity of functional activation patterns as syllable identity, talker identity, and degree of acoustic degradation were parametrically manipulated. The authors found that the left and right temporal cortex—specifically, bilateral clusters encompassing the STG, STS, and MTG—responded similarly to trials where syllable identity and talker identity were held constant, regardless of the kind of acoustic degradation in the speech signal. Thus, the bilateral temporal cortex may specifically support the integration of phonetic identity and talker identity, as changes to either one of these dimensions can affect the pattern of functional activity.

However, it is notable that the stimuli used in these studies were relatively unambiguous, where there might be little pressure to link phonetic tokens to a talker identity. In order to clarify the mechanisms through which phonetic details and talker information are integrated, it may be important to consider the case of phonetically ambiguous stimuli, where listeners must appeal to talker information in order to resolve phonetic identity. Furthermore, the finding that a set of right STS voxels was highly discriminative for both phonetic discrimination and talker discrimination provides a clue that the right STS may be an important interface between a left-lateralized phonetic processing system and a right-lateralized talker processing system.

**Figure 1.**
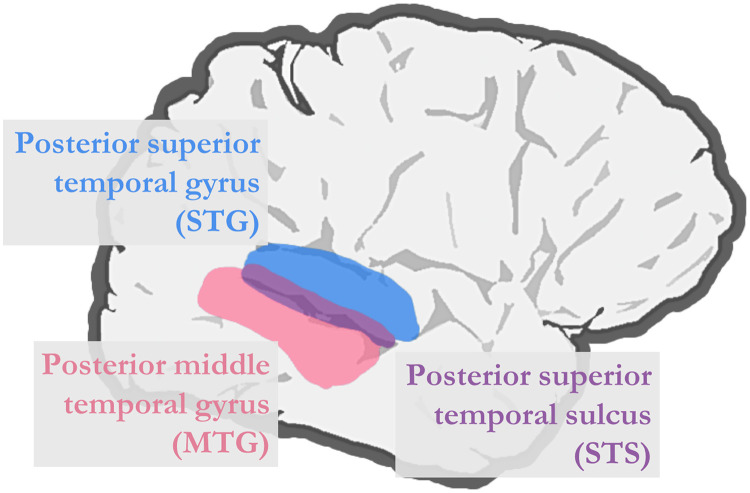
Right temporal brain regions that may support the integration of phonetic detail with talker information. Previous studies have implicated the right posterior STG and MTG, as well as the right STS, which lies between the STG and MTG, in conditioning phonetic identity on talker information. However, the precise contribution of each of the different brain regions remains underspecified.

In concert, the findings from these studies suggest that some set of voxels in the temporal cortex may serve as an interface between the left-lateralized phonetic processing system and the right-lateralized talker processing system. However, there are a number of important details that need to be clarified. While the results of Formisano et al. (2008) implicate the STS in particular, the results of Evans and Davis (2015) suggest that the STG and MTG may also contribute meaningfully. As such, an important next step is to more precisely characterize the contributions of the STG, STS, and MTG in integrating phonetic detail with talker information (Figure 1).

In addition to the possibility that phonetic information and talker information are simultaneously represented in some part of the bilateral temporal lobes (i.e., that there is some degree of *overlap* between the phonetic processing system and the talker processing system), it is also possible that talker-specific phonetic processing is partly achieved through functional connections between the left and right temporal cortex (i.e., that there is some degree of *interaction* between the two neural systems). Evidence that the integration of phonetic information and talker information is supported by interhemispheric interactions comes from a study by von Kriegstein et al. (2010). In that study, participants listened to a series of syllables produced by talkers who differed in vocal tract length. Vocal tract length is a parameter that affects the formants (i.e., the frequency bands where acoustic energy is most concentrated) in the speech signal, and as such, it can systematically influence the acoustic information associated with different phonemes (Johnson, 2008; Joos, 1948) as well as different talkers (Fitch & Giedd, 1999). Strikingly, von Kriegstein et al. found that the response of the right posterior STG/STS was influenced by vocal tract length, but only when listeners were engaged in a speech perception task (a one-back syllable monitoring task) and not in a control task (one-back loudness monitoring or one-back talker monitoring). The authors suggested that the right posterior STG/STS activity might reflect the estimation of vocal tract length for the purpose of talker normalization; that is, the recruitment of the right STG/STS might underlie the process by which listeners leverage their knowledge of the talker’s formant structure to make adjustments to the mapping from acoustics to phonetic categories. This finding implicates the right STG/STS in the integration of phonetic detail and talker information. However, the authors also found a cluster of voxels in the left STG that was sensitive to vocal tract length, and critically, the activity of this left STG cluster was strongly correlated with the activity of an analogous STG cluster in the right hemisphere during the speech recognition task; that is, the two regions were functionally connected. Notably, the functional connectivity between the left and right STG was significantly weaker when listeners were performing the control tasks. Taken together, the data from von Kriegstein et al. suggest that in order to leverage talker information for the purposes of speech perception, listeners rely on both the left and right temporal cortex. That is, the integration of talker detail and phonetic information may be partly supported by interactions between right hemisphere regions associated with talker processing and left hemisphere regions associated with phonetic processing.

The goal of the current fMRI study was to provide a more precise characterization of the neural mechanisms by which listeners contact talker-specific generative models. We specifically sought to examine the (implicit) beliefs that form over the course of lexically guided perceptual learning; in doing so, we hoped to better characterize the neural systems that listeners use when updating their beliefs about how different talkers produce their speech sounds. We considered two possible neural mechanisms. First, we considered the possibility that phonetic detail and talker information are simultaneously encoded in a single brain region, representing an overlap between the neural system for phonetic processing and the neural system for talker processing. Here, we focused specifically on the right temporal cortex, which previous studies have suggested may support the integration of talker information and phonetic detail (Evans & Davis, 2015; Formisano et al., 2008). Second, we considered the degree to which talker-specific phonetic processing is achieved by the coordinated activity of the left and right temporal cortex—that is, by functional interactions between the two neural systems. Importantly, these two mechanisms are not mutually exclusive, and the process of leveraging talker information for phonetic processing may depend on both.

The approach of our study was as follows. Over the course of several exposure runs, listeners were exposed to two talkers, one of whom produced an ambiguous fricative (a blend between /s/ and /∫/) in /s/-biased contexts like *epi?ode* and the other of whom produced the same ambiguous fricative in /∫/-biased contexts like *friend?ip*. Thus, in these runs, lexical information would encourage listeners to form talker-specific beliefs of how acoustic-phonetic information maps onto the /s/ and /∫/ sounds. In test runs, listeners heard stimuli from a /s/-/∫/ continuum and performed a phonetic categorization task. Critically, to interpret ambiguous phonemes, listeners had to condition phonetic identity on talker information (talker identity or acoustic patterns that covary with talker). Neuroimaging data were collected using fMRI. To assess whether any region integrates phonetic information and talker information, we conducted a series of searchlight analyses to identify voxels where the local pattern of activation contained information about the phonetic identity of the test stimulus as well as about the talker who produced it. To measure the extent to which phonetic information and talker information are integrated through interactions between distinct brain areas, we conducted a functional connectivity analysis. The stimuli and analysis code for this work are publicly available at https://osf.io/6j7nr/.

We first performed a behavioral experiment (Experiment 1) aimed at confirming that listeners could simultaneously maintain distinct sets of beliefs for our two talkers. Once we established that this was the case, we conducted an fMRI experiment (Experiment 2) aimed at clarifying the contributions of different neural regions when listeners must condition phonetic identity on talker information.

## EXPERIMENT 1

Previous lexically guided perceptual learning studies have established that listeners are able to maintain distinct sets of beliefs for two different talkers (e.g., one who produces an ambiguous /s/-/∫/ blend in /s/-biased contexts like *epi?ode* and one who produces an ambiguous /s/-/∫/ blend in /∫/-biased contexts such as *friend?ip*; Kraljic & Samuel, 2007; Luthra et al., 2021). In Experiment 1, we sought to verify that we could induce talker-specific perceptual learning, adapting the approach of Luthra et al. (2021) to meet the design constraints of our fMRI experiment (Experiment 2). Thus, Experiment 1 provides a baseline characterization of the learning effects that we would expect to observe in Experiment 2.

### Materials and Methods

#### Stimuli

Stimuli were taken from Luthra et al. (2021), to which the reader is directed for additional information on stimulus construction. In brief, the stimuli consisted of a set of items for exposure runs and a separate set of items for test runs. The exposure items consisted of 32 words, 16 containing a word-medial /s/ and 16 containing a word-medial /∫/. These two sets of items were matched in word frequency, number of syllables prior to the fricative, and number of total syllables. A female native speaker of American English produced lexically consistent (e.g., *episode*) and lexically inconsistent (e.g., *epishode*) versions of each item. Word-nonword continua were made for each item using STRAIGHT (Kawahara et al., 2008), and for each item, the authors selected a continuum step with an unambiguous fricative (e.g., *episode*, *friendship*) and a step with an ambiguous fricative (*epi?ode*, *friend?ip*). Male versions of these stimuli were created by applying the “Change Gender” function in Praat (Boersma & Weenik, 2017), which uses a Pitch Synchronous Overlap and Add (PSOLA) algorithm to shift the pitch of a stimulus and adjust the formant ratio; the pitch change captures the general phenomena that male voices tend to have more massive vocal folds and thus lower pitch (e.g., Slavit, 1999), whereas the formant ratio is related to vocal tract length, which is in turn correlated with body size (e.g., Fitch & Giedd, 1999). For the test phase, the female talker was recorded saying the words *sign* and *shine*; a 7-step continuum was created in STRAIGHT, and male versions of the stimuli were created using the “Change Gender” function as described above.

For the present experiment, we opted to use only four steps from each test continuum, following the approach of Myers and Mesite (2014). Pilot testing with these stimuli indicated that steps 1 and 6 were relatively unambiguous, with participants interpreting the initial segment as /∫/ 2% and 98% of the time, respectively. Steps 3 (34% /∫/ response) and 4 (76% /∫/) were selected as the ambiguous step, and both steps were associated with sizable shifts in perception based on previous exposure.

#### Participants

Twenty-six individuals were recruited using the online participant recruitment platform Prolific (Prolific Academic, 2022). All participants were English-speaking monolinguals residing in the United States between the ages of 18 and 34. All participants had normal or corrected-to-normal vision, no hearing loss, and no language-related disorders. Individuals who had participated in previous studies using these stimuli were not eligible for the current experiment, and only participants using a desktop computer were able to complete the experiment. All participants provided informed consent, and each participant was paid $7.50 for their time. All procedures were approved by the University of Connecticut Institutional Review Board.

Data from three participants were excluded due to technical errors. We decided a priori to exclude the data of any participants who failed the headphone screening test (described below) twice, resulting in the exclusion of one more participant. Luthra et al. (2021) also excluded participants who failed to respond to 10% or more of the trials in either the exposure or test runs as well as participants who showed less than 70% accuracy in classification of the unambiguous endpoints during the phonetic categorization task. Applying this criterion led to the exclusion of two additional participants. After these exclusionary criteria were applied, data from 20 participants (16 female, 4 male; mean age: 26, age range: 20–31) were included in the analyses for Experiment 1.

#### Procedure

After providing informed consent, participants completed a screening test designed to verify that they were using headphones (Woods et al., 2017). In this test, a sequence of auditory tones is presented binaurally, and participants are asked to indicate which tone is quietest. Critically, one tone in the sequence is presented in anti-phase across the stereo channels. When presented over loudspeakers, waveforms from the left and right channels cancel centrally, resulting in an attenuated signal; thus, participants completing this task over loudspeakers generally tend to select this tone. Over headphones, however, the anti-phase tone is not heard as the quietest. Thus, participants are expected to perform differently depending on whether they are wearing headphones, and this can be used to screen participants thought to be using their computer’s loudspeakers. After completing the headphone screening test, participants answered a short set of demographics questions and then moved on to the main experimental task.

**Figure 2.**
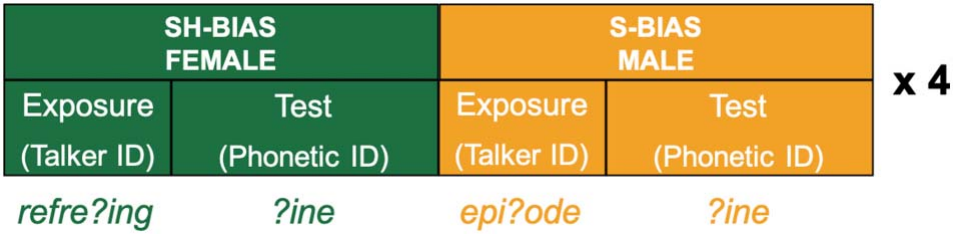
Overview of the design for Experiments 1 and 2. Listeners alternated between exposure runs (during which they performed a talker identification task) and test runs (during which they performed a phonetic categorization task). During an exposure run, listeners would hear one of the two talkers (e.g., the female talker) producing ambiguous fricatives (noted /?/) in lexically biased contexts (e.g., /∫/-biased contexts like *friend?ip*). During a subsequent test run, listeners heard stimuli from a *sign*–*shine* continuum, with all items produced by the same talker as in the previous exposure run. Listeners completed a total of 16 experimental runs. We counterbalanced which talker was associated with which biasing condition as well as which talker listeners heard first.

As schematized in Figure 2, the experimental task alternated between exposure runs and test runs. During each exposure run, listeners heard speech from one of the two talkers, and heard ambiguous fricatives from that talker in lexical contexts that led to the interpretation as either /s/ or /∫/, with the particular bias of the talker (i.e., whether the male or female talker was /s/- or /∫/-biased) counterbalanced across participants. For instance, if the talker was /s/-biased, the listener would hear 16 ambiguous fricatives in contexts where lexical information biased them to interpret the segment as /s/ (e.g., *epi?ode*) and 16 clear productions of the contrastive category (e.g., *refreshing* with a clear /∫/). In test runs, listeners performed a phonetic categorization task with stimuli from a *sign*–*shine* continuum. In each test run, stimuli were produced by the same talker who had produced the stimuli during the previous exposure run, and listeners heard 12 instances of each of the four steps (two unambiguous, two ambiguous). After performing an exposure run and a test run with one talker, listeners would perform an exposure run and a test run with the other talker. This set of four runs was repeated four times over the course of the experiment, for a total of 16 runs. The order of the talkers (male first or female first) was also counterbalanced across participants.

During exposure trials, listeners simply had to indicate whether the talker they were hearing was a male talker or a female talker. Critically, because talker was held constant in each run, participants made the same response for the entirety of each exposure run. Our goal in adopting such a simple task was to consistently remind listeners of the association between the phonetic information and the talker, potentially encouraging the formation of talker-specific generative models. Notably, lexically guided perceptual learning has been observed with a variety of exposure tasks, several of which have required relatively shallow processing (Drouin & Theodore, 2018; Eisner & McQueen, 2006; Luthra et al., 2021; Maye et al., 2008; McQueen et al., 2006; White & Aslin, 2011). Furthermore, previous work has suggested that attending to talker identity encourages listeners to encode talker-specific phonetic detail (Goldinger, 1996; Theodore et al., 2015). Finally, previous research has demonstrated that the degree to which right temporal regions respond to speech is modulated by the extent to which listeners attend to talker identity (Schall et al., 2014; von Kriegstein et al., 2003), motivating the use of a talker identification task for the current study.

Because the overall goal of the present study was to investigate the neural mechanisms through which listeners contact talker-specific generative models, we are particularly interested in characterizing neural activity during the test phase, during which listeners must leverage talker information to perform phonetic classification. As such, we wanted to have a relatively large number of test trials. However, because listeners continually update their beliefs of how a particular talker produces their speech sounds (Saltzman & Myers, 2021; Tzeng et al., 2021), we were concerned that hearing unambiguous fricatives during a protracted test phase might encourage listeners to abandon the beliefs formed during the exposure phase. It was for this reason that we opted to have listeners in this study alternate between exposure runs and test runs, following the approach of Myers and Mesite (2014). In this way, a listener’s beliefs about how the talker produced their speech sounds would be re-established prior to each test phase. Note that in theory, listeners could show what might appear to be talker-specific learning if they just adjusted their category boundary based on the most recent exposure condition, without actually forming talker-specific beliefs; however, results from previous lexically guided perceptual learning studies with multiple voices suggest that listeners do establish and maintain talker-specific beliefs for multiple talkers, rather than simply being guided by their most recent exposure block (Kraljic & Samuel, 2007; Luthra et al., 2021).

Across participants, we counterbalanced which talker listeners heard first (the female or the male) as well as the bias (/s/-bias or /∫/-bias) assigned to each talker. Within each run, trial order was randomized for each participant, and response mappings were counterbalanced across participants. Participants had 4 s to respond on each trial, and each trial was followed by a 1 s intertrial interval. Experiment 1 was programmed using the Gorilla online experiment builder (Anwyl-Irvine et al., 2020).

#### Analyses

Trial-level phonetic categorization data were analyzed with a mixed-effects analysis implemented in R (Version 4.2.1; R Core Team, 2022). We specifically used the “mixed” function in the afex package (Singmann et al., 2018); this function implements mixed-effects models using the “glmer” function of the lme4 package (Bates et al., 2015) and assesses the significance of each fixed effect using likelihood ratio tests. We specified a logit link for our analyses, as appropriate for dichotomous data.

Our model attempted to predict the likelihood of a subject making a “shine” response and considered fixed factors of Step (scaled), Bias (sum-coded [1, −1], sh-bias, s-bias), and Talker (sum-coded [1, −1], female, male). The model also included random intercepts for each subject, random by-subject slopes for Step, Bias, and Talker, and random by-subject interactions between Step and Bias as well as between Step and Talker. (Because Bias and Talker were consistently paired for a given individual—that is, a given listener either heard an /s/-biased female talker or an /∫/-biased female talker, but never both—we did not include random by-subject interactions between Bias and Talker.) Thus, the full model syntax was specified as:

SH_resp ∼ step * bias * talker + (step:bias + step:talker + step + bias + talker | subject)

This represents both the maximal model (Barr et al., 2013) and the most parsimonious, as a backward-stepping procedure indicated that a simpler random effect structure yielded a significantly worse model fit (Matuschek et al., 2017).

### Results

**Figure 3.**
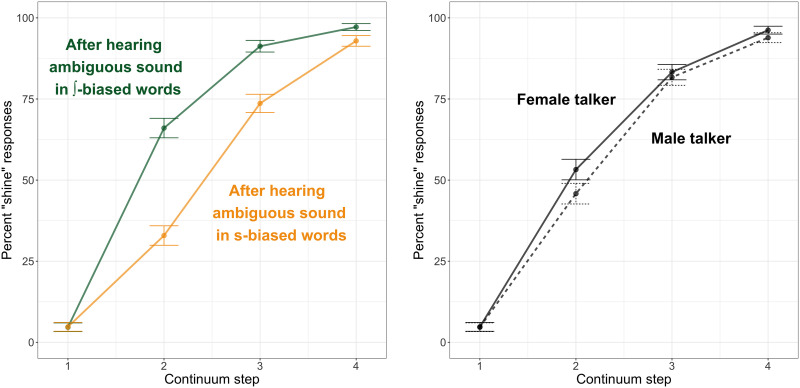
Phonetic categorization data from Experiment 1 (behavioral pilot). In both plots, the *x*-axis indicates the continuum step on the sign–shine continuum, while the *y*-axis indicates the percentage of “shine” responses at that step. Error bars indicate 95% confidence intervals around the mean. The left panel shows the data as a function of whether the talker previously produced ambiguous fricatives in /∫/-biased (green line) or /s/-biased (yellow line) contexts, and the right panel shows the data as a function of the talker’s gender, female (solid line) or male (dashed line).

Phonetic categorization data are plotted in Figure 3. Visually, it appears that listeners’ responses were strongly affected by whether the talker had previously produced the ambiguous sound in /∫/-biased or /s/-biased contexts (left panel) but not strongly affected by the gender (male or female) of the talker (right panel).

Results of the statistical analysis are given in Table 1. We observed an expected main effect of Step, as participants made more “shine” responses when presented with steps closer to the “sh” end of the continuum. There was a significant main effect of Bias, with participants making more “sh” responses if they had previously heard the ambiguous sound in contexts where /∫/ was the lexically consistent phoneme; that is, we observed lexically guided perceptual learning. There was also a marginal Step × Bias interaction (*p* = 0.06), driven by a slightly larger effect of the biasing context at intermediate (ambiguous) continuum steps. Finally, we observed a significant Step × Talker interaction; while differences between the two talkers were minimal at most continuum steps, there was a slight difference at Step 2, with participants more likely to make a “shine” response for the female talker (mean: 0.53, *SE*: 0.02) than for the male talker (mean: 0.46, *SE*: 0.02), regardless of whether the talker produced ambiguous fricatives in /s/-biased or /∫/-biased contexts during the exposure phase. No other effects were significant.

**Table 1.** Analysis of Experiment 1 phonetic categorization data (behavioral pilot)

Fixed effect	χ^2^(1)	*p* value	
Step	30.02	<0.0001	***
Bias	15.6	<0.0001	***
Talker	0.23	0.63	
Step × Bias	3.65	0.06	+
Step × Talker	5.54	0.02	*
Bias × Talker	0.57	0.45	
Step × Bias × Talker	0.72	0.40	

*Note*. *** indicates *p* < 0.001, ** indicates *p* < 0.01, * indicates *p* < 0.05, and + indicates *p* < 0.10.

### Discussion

In Experiment 1, listeners demonstrated talker-specific perceptual learning for two talkers (one female, one male); for one talker, an ambiguous fricative corresponded to the phoneme /s/, while for the other talker, the same ambiguous fricative corresponded to /∫/. Notably, talker-specific learning was observed with a relatively long session, in contrast to previous studies that have observed talker-specific learning in relatively short sessions (Kraljic & Samuel, 2007; Luthra et al., 2021). In a follow-up analysis, we tested whether the size of the learning (Bias) effect depended on which set of four runs (first, second, third, or fourth; see Figure 2) the participant was completing. Specifically, our statistical model tested for fixed effects of Set (scaled) in addition to the factors used in our initial analysis (Step, Bias, and Talker). We included each of the random effect terms used in the initial analysis as well their interactions with Set. We observed significant fixed effects of Step and Bias as well as a significant Step × Bias interaction. No other effects were significant. The lack of any significant interactions between Bias and Set suggests that the size of the learning effect was constant across the experimental session. Thus, Experiment 1 showed that listeners can form separate beliefs for how two talkers produce their speech sounds and can maintain these beliefs over a relatively long experimental session. Having observed robust learning effects in this experiment, we were able to conduct an fMRI experiment (Experiment 2) to probe the neural mechanisms through which listeners contact talker-specific beliefs for how acoustic-phonetic information maps onto phonetic categories.

## EXPERIMENT 2

In Experiment 2, participants performed the same task as in Experiment 1 but did so in the scanner while fMRI data were collected. Thus, listeners were exposed to two talkers, one of whom produced an ambiguous fricative (a /s/-/∫/ blend) in place of a clear /s/ and one of whom produced the ambiguous fricative in lieu of /∫/; during exposure runs, phoneme identity was disambiguated through lexical information (e.g., *epi?ode*). After each exposure run, listeners performed a phonetic categorization task with a *sign*–*shine* continuum produced by the talker they had heard during the previous run. Critically, to resolve the phonetic identity of each test stimulus (i.e., those presented in the phonetic categorization task), listeners must leverage talker information; this is particularly the case for the ambiguous stimuli, since for one talker an ambiguous fricative corresponds to /s/, but for the other it corresponds to /∫/. As such, a consideration of the functional activation during the phonetic categorization task can clarify the neurobiological mechanisms through which listeners condition phonetic identity on talker information.

First, we consider both the possibility that some set of regions represents both talker information and phonetic information. The extant literature has suggested that the posterior temporal cortex may play such a role, though the specific contributions of regions within posterior temporal cortex (e.g., STG, STS, MTG) remain unclear, as do the specific contributions of the right and left hemispheres (Evans & Davis, 2015; Formisano et al., 2008; Luthra et al., 2020; Myers & Mesite, 2014). The approach of the current study was to conduct a series of searchlight analyses, allowing us to identify voxels that are important for classification based on talker information as well as voxels that contribute to phonetic classification; of interest is the overlap between these sets of voxels, as these regions potentially constitute an integration site for talker information and phonetic information.

A core methodological feature of the current study is the use of searchlight analyses (Kriegeskorte et al., 2006). In this approach, classification analyses are performed within a roving “searchlight,” and performance within a searchlight is assigned to the central voxel. Thus, searchlight analyses only consider the pattern of activation within spatially adjacent voxels. This is in contrast to previous work by Formisano et al. (2008), who also attempted to classify functional activation patterns based on talker and phonetic information but whose approach involved the use of recursive feature elimination (RFE; De Martino et al., 2008). Specifically, Formisano et al. trained their classifier on the patterns of activation across all the voxels in a prescribed region (the bilateral temporal cortex) and then used RFE to identify which voxels contributed most to classification. RFE can be particularly useful when the patterns of interest are distributed across a broad set of regions.

One potential concern relates to the spatial specificity of searchlight analyses—because performance within a searchlight is assigned to the central voxel, classification is undoubtedly also supported by surrounding voxels. Similarly, in RFE, because the machine learning algorithm has access to a relatively large set of voxels for performing classification, it is not necessarily the case that accurate classification can be performed using only the most discriminative voxels (as identified through RFE). That is, even if RFE can identify the set of voxels that are most informative for classification, voxels outside of this most discriminative set may still be necessary for accurate classification. For both types of MVPA, it is critical to consider the contributions of a voxel with respect to the full set of voxels under consideration. Because searchlight analyses consider only the local pattern of activation (as opposed to RFE, where the contributions of an identified voxel may be influenced by nonlocal voxels) and because a primary aim of the current study was to more precisely characterize the contributions of particular subregions in temporal cortex (STG, STS, and MTG), we opted to use a searchlight approach.

In addition to testing whether the integration of talker and phonetic information is achieved in a single region, we also considered the possibility that this integration is achieved through the coordinated activity of multiple regions. That is, there may be one set of regions that represents talker information and a distinct set of regions that represents phonetic information, and the two sets of regions may work together to achieve the integration of talker and phonetic detail. Since talker processing tends to rely relatively strongly on the right hemisphere and phonetic processing tends to rely more strongly on the left hemisphere, this would likely be achieved specifically through functional connections between the left and right hemisphere. To assess this possibility, we first identified which voxels in the right hemisphere are sensitive to talker information (using a searchlight analysis, as above); we then tested whether the activity of these voxels predicts the activity of other brain regions during the phonetic classification task. Of interest was whether this latter connectivity analysis would identify left hemisphere regions implicated in phonetic processing—for instance, those in the left temporal cortex (e.g., STG and MTG; Blumstein et al., 2005; Desai et al., 2008; Scott et al., 2000), left parietal cortex (e.g., the supramarginal gyrus and angular gyrus; Blumstein et al., 2005; Lee et al., 2012; Raizada & Poldrack, 2007) or left frontal cortex (especially the left inferior frontal gyrus, or IFG; Blumstein et al., 2005; Lee et al., 2012; Rogers & Davis, 2017; Xie & Myers, 2018).

### Experimental Design

#### Stimuli

Experiment 2 used the same stimuli as in Experiment 1.

#### Participants

Twenty individuals (14 female, 6 male; mean age: 21, age range: 18–28) were recruited from the University of Connecticut community. This sample size is consistent with previous MVPA studies (Correia et al., 2015; Feng et al., 2019; Joanisse et al., 2007; Kilian-Hütten et al., 2011; Lee et al., 2012; Luthra et al., 2020), which typically have an average sample size of about 17 (range: 10–30). All participants were right-handed native speakers of North American English and indicated that this was the only language they spoke prior to age 13. Participants did not have any hearing deficits, had normal or corrected-to-normal vision, had no history of neurological impairment, and met all MRI safety requirements (e.g., no ferromagnetic material in their bodies). Participants provided informed consent, and each participant was paid $50 for their time. All participants met the data inclusion criteria used in Experiment 1 (responding to more than 90% of the trials in both the exposure and test runs, showing at least 70% accuracy in phonetic classification of the unambiguous endpoints). All procedures were approved by the University of Connecticut Institutional Review Board.

#### Procedure

**Figure 4.**
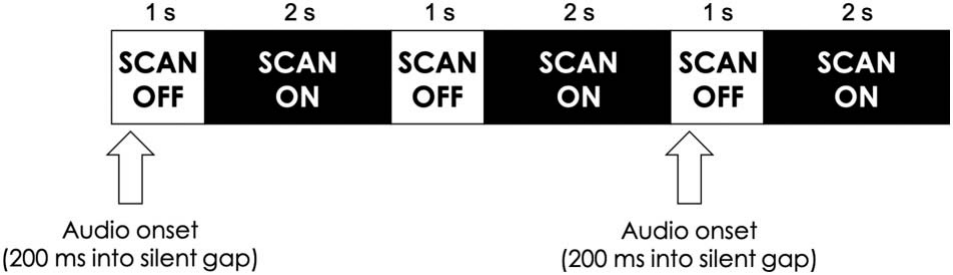
Clustered volume acquisition design for Experiment 2. On critical trials, auditory stimuli were presented in 1 s gaps between each 2 s scan period. Silent trials were interspersed between critical trials, allowing us to jitter the onsets of critical trials and thereby model the hemodynamic response appropriately.

While the procedure of Experiment 2 was essentially the same as that of Experiment 1, slight timing changes were made to the design to make the experiment compatible with fMRI. As in Experiment 1, participants alternated between exposure (talker identification) runs and test (phonetic categorization) runs, completing 32 trials (16 ambiguous, 16 unambiguous) in each exposure run and 48 trials (12 repetitions at each of the four continuum steps) in each test run. Auditory stimuli were presented in silent intervals between scans (Edmister et al., 1999). As shown in Figure 4, each trial consisted of a 1 s “scan off” period, during which the auditory stimulus was presented, with the onset of the stimulus falling 200 ms into the silent interval; functional images were collected during a subsequent 2 s scan, during which a fixation cross was displayed on the screen. To appropriately model the hemodynamic response in each condition, each run included an equal number of “silent” trials as critical trials. Silent trials consist of a period of timing identical to a critical trial, except no stimulus is presented; these silent trials were interspersed between critical trials, thereby allowing us to jitter the onsets of critical trials. The specific order of these trials was determined using the optseq2 program (Greve, 2002). The response mappings were shown on the screen for the first 1,500 ms of each critical trial, and a fixation cross appeared on the screen during the second half of each critical trial as well as throughout each silent trial. Experiment 2 was programmed using the OpenSesame experiment builder (Mathôt et al., 2012).

MRI data were collected at the University of Connecticut Brain Imaging Research Center using a 3T Siemens Prisma scanner (Siemens Medical Solutions, 2022) with a 64-channel head coil. Anatomical images were acquired using a T1-weighted magnetization-prepared rapid acquisition gradient echo (MPRAGE) sequence (TR = 2,400 ms, TE = 2.15 ms, FOV = 256 mm, flip angle = 8°) with 1 mm sagittal slices. Axial-oblique functional echo planar images were acquired using a T2*-weighted sequence in ascending, interleaved order (TR = 3.0 s [effective TR of 2.0 s with a 1.0 s delay], TE = 25 ms, 52 slices, 2.5 mm thickness, in-plane resolution = 2 mm × 2 mm, FOV = 220 mm, flip angle = 62°, multiband acceleration factor = 2). In total, 64 volumes were acquired for each exposure run, and 96 volumes were acquired for each test run. The entire MRI session lasted approximately 90 min.

Stimuli were presented using a transducer system (Silent Scan SS-3100 made by Avotec (n.d.)) coupled to a pair of insert headphones (Conformal Headset made by Avotec (n.d.)). With this system, auditory stimuli can be delivered through two flexible tubes that penetrate foam ear tips. The ear tips are rolled down before they are placed in the participant’s ear canal, where they then expand. In this way, the insert headphones are intended to provide hearing protection from the scanner noise while also allowing auditory stimuli to be delivered to the participant.

### In-scanner Behavior

#### Behavioral results

Behavioral data were analyzed following the same approach as in Experiment 1. We used the same model structure as in Experiment 1; as before, the maximal random effects structure was also the most parsimonious.

**Figure 5.**
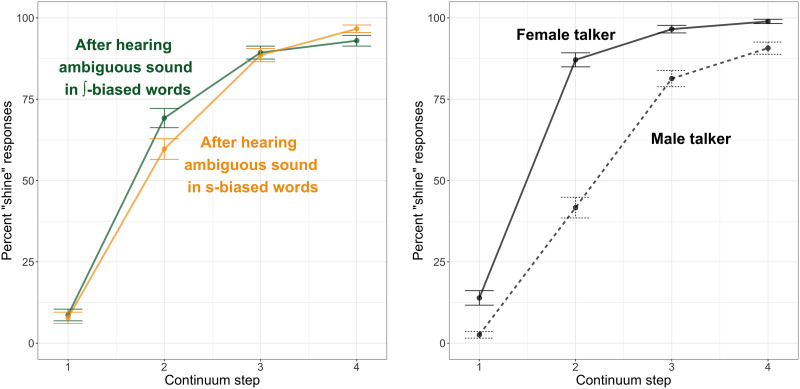
Phonetic categorization data from Experiment 2 (fMRI experiment). In both plots, the *x*-axis indicates the continuum step on the *sign*–*shine* continuum, while the *y*-axis indicates the percentage of “shine” responses at that step. Error bars indicate 95% confidence intervals around the mean. The left panel shows the data as a function of whether the talker previously produced ambiguous fricatives in /∫/-biased (green line) or /s/-biased (yellow line) contexts, and the right panel shows the data as a function of the talker’s gender, female (solid line) or male (dashed line).

Phonetic categorization data are plotted in Figure 5. In contrast to the results of Experiment 1, there appears to be a minimal effect of Bias in our fMRI participants, with only a slight difference in the categorization functions of listeners who heard the ambiguous fricatives in /∫/-biased contexts compared to /s/-biased frames (left panel). However, listeners appeared to differ dramatically in how they categorized stimuli produced by the female talker as compared to the male talker (right panel).

Results of the statistical analysis are provided in Table 2. We observed the expected effect of Step, with participants making more “sh” responses for stimuli that were closer to the “shine” end of the two continua. However, in contrast to Experiment 1, we did not observe any significant effects of Bias (all *p* ≥ 0.15). Instead, we observed a main effect of Talker, with participants making more “sh” responses for the female talker (mean: 0.74, *SE*: 0.01) than for the male talker (mean: 0.54, *SE*: 0.01). We also observed a significant Step × Talker interaction, as there was a pronounced difference in how often participants made a “sh” response for the female talker (mean: 0.87, *SE*: 0.01) compared to the male talker (mean: 0.41, *SE*: 0.01) at Step 2 in particular.

**Table 2.** Analysis of Experiment 2 phonetic categorization data (fMRI experiment)

Fixed effect	χ^2^(1)	*p* value	
Step	37.05	<0.0001	***
Bias	2.1	0.15	
Talker	25.86	<0.0001	***
Step × Bias	0.76	0.38	
Step × Talker	5.38	0.02	*
Bias × Talker	0.22	0.64	
Step × Bias × Talker	0.46	0.5	

*Note*. *** indicates *p* < 0.001, ** indicates *p* < 0.01, * indicates *p* < 0.05, and + indicates *p* < 0.10.

### Headphone Evaluation

One possible source of the discrepancy in the behavioral results of our two experiments might be the particular headphones that were used. Recall that because Experiment 1 was conducted online, participants used their own headphones, and a psychometric screener was used to ensure participants were using headphones instead of their computer loudspeakers. By contrast, stimuli for Experiment 2 were delivered through a pair of MRI-compatible insert headphones (with sound delivered via flexible tubes that penetrate foam earplugs). Note that because /s/ and /∫/ are distinguished primarily by spectral properties in relatively high frequency ranges, if key spectral information was not delivered faithfully to participants in Experiment 2, then their categorization of our stimuli could have been affected. Thus, before presenting the MRI results of Experiment 2, we first consider the possibility that the headphone setup used for Experiment 2 may have contributed to the unexpected pattern of behavioral results.

There were two other minor differences in design between the two experiments, though we do not believe either of them would have contributed to the discrepancy in the pattern of results across the two experiments. In Experiment 2, stimuli were provided in silent intervals between periods of scanning. Though the scan sequence produces a considerable amount of auditory noise, the stimuli were presented 200 ms after the offset of the scanner noise, so we do not expect that perception of the speech stimuli would have been affected by energetic masking effects. We further note that in order to appropriately model the hemodynamic response, the timing of the trials in Experiment 2 was different from that of Experiment 1. A small number of studies have considered the role of timing in lexically guided perceptual learning, with research suggesting that disambiguating information needs to be encountered in advance of or shortly after a phonetically ambiguous segment (Jesse, 2021; Jesse & McQueen, 2011). However, to our knowledge, there is no evidence that the specific amount of time between trials can influence the degree or pattern of perceptual learning.

To directly assess the impact of the insert headphones, we coupled the insert headphones to an audio recorder and recorded the stimuli, allowing us to approximate the experience of participants in the scanner. We compared the spectral information in these recorded stimuli to the spectral information in the original stimuli.

#### Method

Stimuli were recorded in the MRI control room of the University of Connecticut Brain Imaging Research Center. Stimuli were presented using the same transducer system (the Avotec SS-3100 Silent Scan Audio System) and insert headphones (the Avotec Conformal Headset) as above. Recall that each insert headphone penetrates a foam ear tip. We trimmed a foam ear tip using a pair of scissors (taking care not to cut the headphone tubing itself), rolled the remaining foam, and inserted into in a Larson Davis AEC202 coupler (Larson Davis, 2021) for insert earphones, where the foam tip expanded to fill the coupler. The coupler was connected to a half-inch condenser microphone and preamplifier on a Larson Davis 824 sound level meter (Larson Davis, 2021). Finally, the sound level meter was connected to a Roland R-05 WAV recorder (Roland, 2022), using the AC-1/AC-2 output setting on the sound level meter.

Stimuli were analyzed using Praat (Boersma & Weenik, 2017). We used a spectral subtraction procedure (Boll, 1979), implemented through the “Remove Noise” function in Praat, to eliminate noise introduced by the recording procedure. Noise was defined as the spectral information present during a silent part of the recording (before stimuli began playing). Fricative onsets and offsets were marked visually by the first author. Spectral means (in Hz) were measured from the extracted fricative segments. To characterize the perceptual impact of frequency changes introduced by the headphones, we also report results on a perceptual (Mel) scale. Mel values were computed following the definition provided by Fant (1968):

$$Mel=\frac{1000}{\log2}\log\left(1+\frac{\text{frequency}}{1000}\right)$$

#### Results

**Figure 6.**
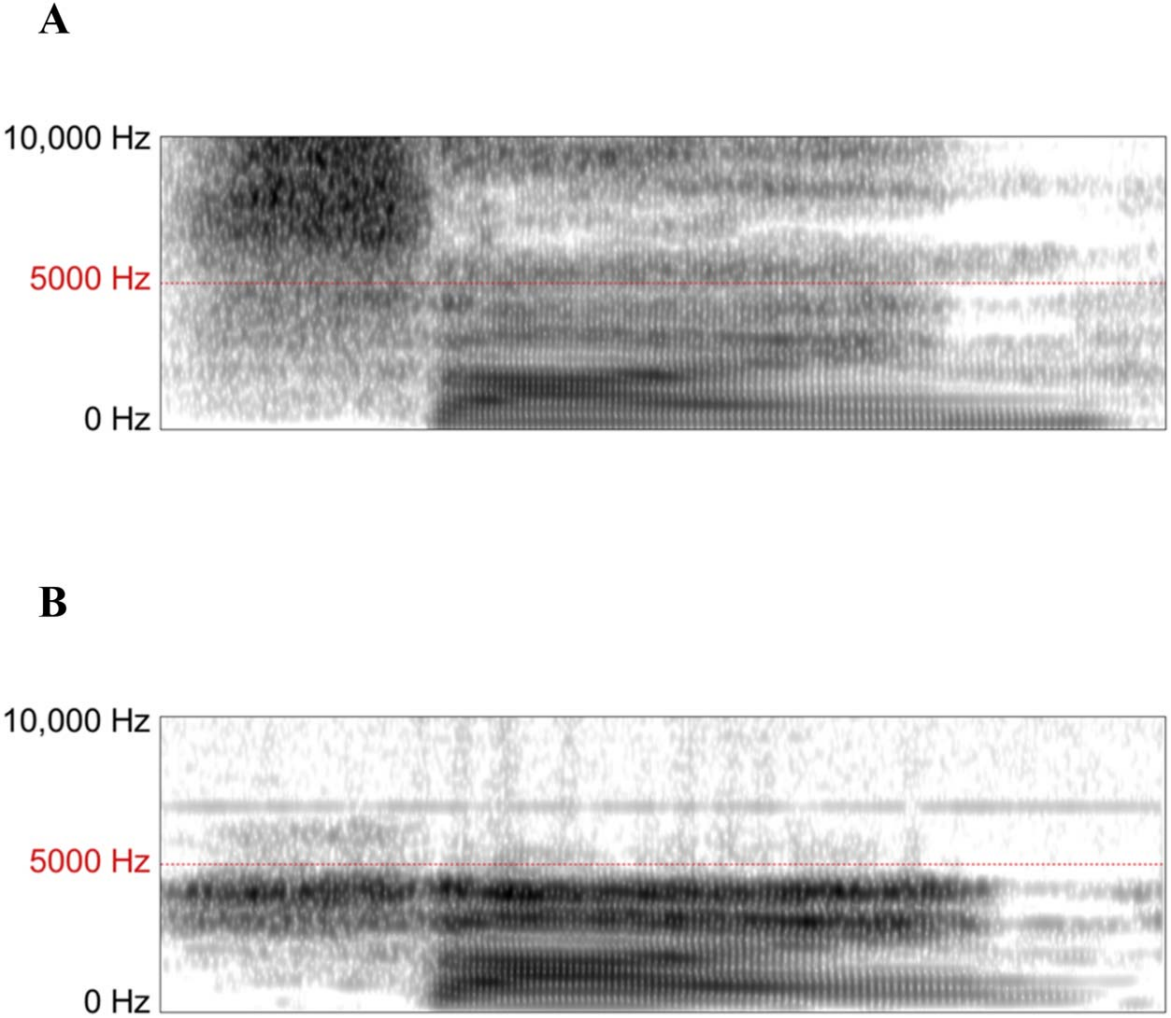
Sample spectrograms for a clear production of “sign” by our female talker (Step 1 of the *sign*–*shine* continuum). A. The spectrogram corresponds to the original stimulus; the spectral center of the fricative /s/ is 9048 Hz. B. The spectrogram corresponds to the stimulus recorded through the insert headphones used in the scanner and therefore approximates the spectral information delivered to our MRI participants. Frequencies above 5000 Hz are heavily attenuated, and the new spectral center of the /s/ fricative is 3950 Hz.

Spectrograms for a sample stimulus (a clear production of “sign” spoken by the female talker, taken from Step 1 of the female test continuum) are provided in Figure 6. The original stimulus (Figure 6A) is characterized by strong amplitudes in the ∼9000 Hz range during the period of frication. However, these frequencies were likely attenuated for our in-scanner participants, as shown in the spectrogram of the stimulus recorded from the insert headphones (Figure 6B).

**Figure 7.**
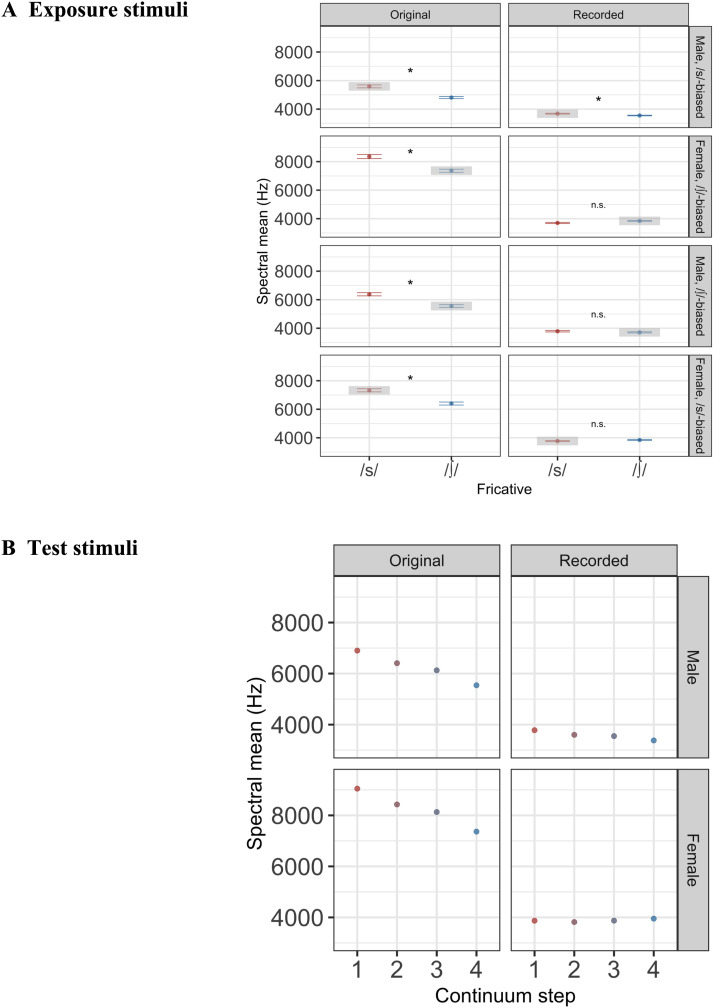
Spectral centers of gravity (in Hz) for the fricatives presented during exposure and test blocks. To assess the impact of the insert headphones used in the scanner, we compared the spectral properties of the original stimuli to those of the stimuli recorded through the insert headphones used in the scanner. Critically, /s/ should be associated with a higher spectral mean than /∫/. A. During exposure, one group of participants heard a male talker producing an ambiguous fricative in /s/-biased contexts and a female talker producing an ambiguous fricative in /∫/-biased contexts (top two rows). The other group of participants heard the male talker producing ambiguous fricatives in /∫/-biased contexts and the female talker producing ambiguous fricatives in /s/-biased contexts (bottom two rows). The /s/ fricatives are shown in red and /∫/ in blue. Gray boxes highlight the ambiguous fricative, the identity of which was determined with lexical information. Error bars indicate standard error. A significant difference between the spectral means of the /s/ and /∫/ stimuli is indicated with an asterisk (*), and a nonsignificant difference is indicated with “n.s.” B. During test, listeners heard a continuum from “sign” (red points) to “shine” (blues); intermediate colors indicate ambiguous continuum steps. Rows indicate talker.

The spectral properties of the stimuli are illustrated in Figure 7 (exposure stimuli in Figure 7A, test stimuli in Figure 7B). Figure 7A highlights spectral properties that were critical for driving learning in each group. For instance, for listeners in the /s/-biased group, lexically guided perceptual learning depends on a distinction between ambiguous /s/ segments and unambiguous /∫/ segments. While the spectral means for these stimuli were relatively well separated in the original stimuli (7329 Hz vs. 6405 Hz for the female talker, 5593 Hz vs. 4810 Hz for the male talker), the filtering introduced by the insert headphones meant that the spectral means were much closer together (3771 Hz vs. 3846 Hz for the female talker, 3684 Hz vs. 3553 Hz for the male talker).

Because learning depends on an acoustic difference between /s/ and /∫/ segments, we conducted two-sample one-tailed *t* tests on the spectral means of the /s/ and /∫/ stimuli presented during exposure, separately testing each talker and each biasing condition. That is, for each of the eight panels shown in Figure 7A, we tested whether the /s/ stimuli (red) had significantly higher spectral means than the /∫/ stimuli (blue). We applied a Bonferroni correction for multiple comparisons (alpha = 0.05 / 8 = 0.00625). Results are summarized in Table 3 and shown visually in Figure 7A. For the original stimuli, we found that the spectral means of the /s/ segments were significantly greater than those of the /∫/ segments. However, the insert headphones attenuated this difference: In three out of four cases, the /s/ segments did not have higher spectral means relative to the /∫/ segments for the stimuli recorded from the headphones.

**Table 3.** Results of one-tailed two-sample *t* tests evaluating whether the spectral means were higher for the /s/ stimuli than for the /∫/ stimuli for each combination of Talker and Bias

Talker/Bias	Original	Recorded
Male, /s/-biased	*t*(30) = 6.27, *p* < 0.0001, *	*t*(30) = 3.39, *p* = 0.0010, *
Female, /∫/-biased	*t*(30) = 5.56, *p* < 0.0001, *	*t*(30) = −2.93, *p* = 0.9968, n.s.
Male, /∫/-biased	*t*(30) = 5.74, *p* < 0.0001, *	*t*(30) = 1.26, *p* = 0.1081, n.s.
Female, /s/-biased	*t*(30) = 6.09, *p* < 0.001, *	*t*(30) = −1.46, *p* = 0.9224, n.s.

*Note*. * indicates significance at an alpha of 0.05/8 = 0.00625 (Bonferroni correction for multiple comparisons). Results indicate that the acoustic difference between /s/ and /∫/—a key prerequisite for lexically guided perceptual learning—was disrupted by the insert headphones used in the scanner.

#### Discussion

To better approximate the listening environment of participants in the scanner, we recorded our stimuli as they were played out through the insert headphones and performed an acoustic analysis. We found that frequencies above 5000–6000 Hz were dramatically attenuated by the insert headphones. We suspect that this is the primary cause for the discrepancy in the behavioral data between Experiment 1 and Experiment 2. While participants in Experiment 2 reported hearing instances of /s/ and /∫/, the attenuation of key spectral information inhibited lexically guided perceptual learning. In our analysis of the spectral centers of gravity, we found that the acoustic difference between the /s/ and /∫/ segments (a key ingredient for learning) was disrupted by the headphones.

Recall that the primary aim of this study was to probe the neural systems that allow listeners to access talker-specific generative models and that we were specifically interested in generative models that had been shaped by lexically guided perceptual learning. Because of the way our headphones impacted stimulus delivery, listeners in Experiment 2 were not influenced by the lexically biasing information in the exposure runs. Nevertheless, listeners did show differential phonetic categorization profiles for the two talkers. As such, Experiment 2 can still allow for a fruitful examination of the neural systems supporting access to talker-specific beliefs; we return to this issue in the General Discussion.

### MRI Preprocessing

Preprocessing of the fMRI data was achieved in AFNI (Cox, 1996), with slightly different processing pipelines used for the functional connectivity analyses and the searchlight analyses. For the functional connectivity analyses, oblique functional images were first warped to a cardinal orientation and resampled to isotropic (2 mm × 2 mm × 2 mm) voxels using the 3dWarp command. Next, functional data were registered to the participant’s anatomical data, aligned to the first volume of each functional run to correct for within-run motion using a six-parameter rigid body transform, and warped from the participant’s native space to Talairach space (Talairach & Tournoux, 1988); these three affine transformations were applied simultaneously to the data to minimize interpolation penalties and were implemented using the align_epi_anat.py program built into afni_proc.py. Functional data were then smoothed using a 4 mm full-width half-maximum kernel and scaled to have a mean value of 100. Two participants only completed 12 of the 16 functional runs, and for one of these participants, substantial motion after the sixth run necessitated a second anatomical scan. For the latter participant, the first half of the functional data was registered to the first anatomical data set and the second half was registered to the second anatomical data set, but the preprocessing pipeline was otherwise identical to the one used for the other participants. Following preprocessing, we also conducted univariate analyses of the data (see Supporting Information available at https://doi.org/10.1162/nol_a_00091).

For searchlight analyses, preprocessing involved transforming functional data from an oblique orientation to a cardinal one, registering functional data to the anatomical data, and aligning the functional data to the first volume of the corresponding run. Consistent with the univariate analysis, registration and alignment were performed using a single transformation. Note that for these analyses, data were not transformed to Talairach space, such that searchlight analyses for each participant could be conducted in their native space. However, as described above, one participant moved substantially midway through the experiment, necessitating the collection of a second anatomical scan. For that participant, the 3dFractionize command was used to transform the Talairach-warped functional images (generated as part of preprocessing for the functional connectivity analyses) back to the participant’s native space; the first anatomical scan (in native space) was used as a template for this transformation. In this way, all functional images for this participant were in the participant’s native space and registered to the same anatomical image.

### Searchlight Analyses

#### Methods

##### Feature estimation.

After preprocessing, we estimated voxel-wise beta weights for every phonetic categorization trial, using a least-sum-squares regression approach suited for fast event-related fMRI designs (Mumford et al., 2012). In this approach, a separate regression analysis is performed for every trial, with each regression involving a critical regressor for the trial of interest and a nuisance regressor for all other trials in that condition (here, all other instances of that same continuum step spoken by that same talker). This feature estimation step was implemented using the -stim_times_IM flag in the 3dDeconvolve command and the 3dLSS command.

##### Talker classification.

**Figure 8.**
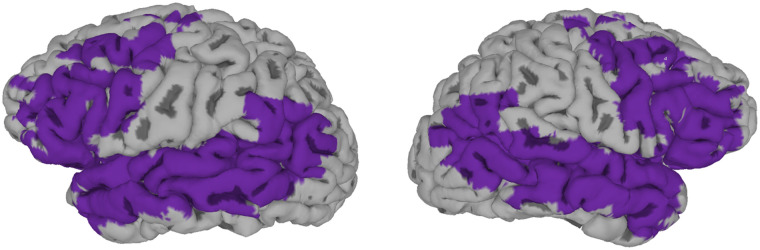
Voxels considered in analyses. For all analyses, we only considered voxels in left-hemisphere regions that have been implicated in language processing as well as the corresponding regions in the right hemisphere. For visualization purposes, volumetric clusters were projected to a surface reconstruction using FreeSurfer (Fischl, 2012) and SUMA (Saad & Reynolds, 2012).

The goal of our first analysis was to identify voxels where the local pattern of activation was informative of talker identity. To this end, a support vector machine was trained on the patterns of activation within an 8 voxel (16 mm) radius searchlight, with activation patterns labeled with respect to talker identity (i.e., female or male). Multivoxel pattern analyses were implemented using The Decoding Toolbox (Hebart et al., 2015). We decided a priori to limit our analyses to anatomical regions that are broadly associated with speech processing—namely, the inferior frontal, middle frontal, insular, superior temporal, transverse temporal, middle temporal, supramarginal, and angular cortices bilaterally, as defined in the AFNI Talairach atlas. This group mask is visualized in Figure 8. We further limited our analyses to voxels that had been imaged in all 20 participants; to achieve this, our group mask was transformed to each subject’s native space using the 3dFractionize command in AFNI (Cox, 1996).

Recall that during the experiment, runs were organized into sets of four, with each set involving an exposure run and a phonetic categorization run for each talker (Figure 2). Cross validation was achieved using a leave-one-set-out approach, such that in each cross-validation fold, the classifier was trained on data from three phonetic categorization runs from each talker (or, for the two participants who did not complete the experiment, on two phonetic categorization runs for each talker) and tested on data from two held-out runs, one for each talker. In this way, there was an equal number of male and female training trials for each cross-validation fold. For each searchlight, the classification performance score at test (accuracy minus chance) was assigned to the central voxel of the searchlight. The support vector machine’s regularization parameter was set at C = 1, and default settings were used for all other parameters.

Subject-level classification maps were converted to Talairach space and blurred using a 4 mm full-width half-maximum kernel in AFNI. The resultant classification maps were then submitted to a one-sample *t* test using the 3dttest++ command. To correct for multiple comparisons, we used a cluster size threshold of 116 voxels (voxel-wise *p* < 0.05, cluster-level α < 0.05).

##### Phonetic classification in regions associated with speech perception.

In a second searchlight analysis, we sought to identify voxels where the local activation pattern was informative of phonetic identity. For this analysis, we considered the same set of voxels that were used in the talker classification searchlight analysis. This analysis considered only trials in which participants heard the unambiguous continuum endpoints, and trials were labeled with respect to the expected response (i.e., Step 1 labeled as /s/ and Step 4 labeled as /∫/). Thus, there was an equal number of trials in each training class. As before, we used an 8 voxel radius searchlight with a leave-one-set-out cross-validation scheme and the same support vector machine parameters as before. By-participant classification maps were converted to Talairach space, blurred using a 4 mm full-width half-maximum kernel, and submitted to a one-sample *t* test, and the same cluster correction was applied to the resultant group map. Of interest is the extent to which this phonetic classification searchlight analysis identified the same voxels as the talker classification searchlight analysis described above.

##### Phonetic classification in regions that support talker classification.

Our final searchlight analysis also sought to identify voxels that were informative of phonetic identity, but in this analysis, we limited our search to the voxels that were identified as showing above-chance accuracy in the talker classification searchlight analysis (rather than testing the full set of regions involved in speech processing, as above). Because these ROIs were defined at the group level, we transformed the functional ROIs to each subject’s native space using the 3dFractionize command in AFNI. For this analysis, we used a smaller (3 voxel) radius searchlight, since the searchlight analysis was being conducted over a smaller set of voxels than before; this also improves the spatial resolution of the analysis, as classification in an individual voxel is supported by a relatively small number of neighboring voxels.

In contrast to the previous phonetic classification searchlight analysis, this analysis considered all phonetic categorization trials (i.e., both ambiguous and unambiguous continuum steps), and activation patterns were labeled based on the participants’ trial-by-trial behavioral responses. To ensure that the classifier was not systematically biased toward whichever response (/s/ or /∫/) a participant made more often, we balanced the number of training trials from each class by subsampling from the more represented class; this balancing procedure was repeated 10 times for each cross-validation fold, and the average of the 10 repetitions was taken as the classifier’s performance for that cross-validation fold. Note that this approach would have been computationally impractical for the previous phonetic classification searchlight analysis, as it would have involved performing 10 repetitions for each cross-validation fold for a relatively large number of voxels (i.e., for all the voxels shown in Figure 8). Here, this approach is tenable because of the relatively small ROIs constraining the searchlight analysis. As before, the support vector machine’s regularization parameter was set at C = 1, and default settings were used for all other parameters. The resultant subject-wise classification maps were warped to Talairach space, blurred using a 4 mm full-width half-maximum kernel, and submitted to a one-sample *t* test. Because a cluster correction had been applied to define the ROIs for this analysis, no further cluster correction was applied at this point.

#### Results

**Figure 9.**
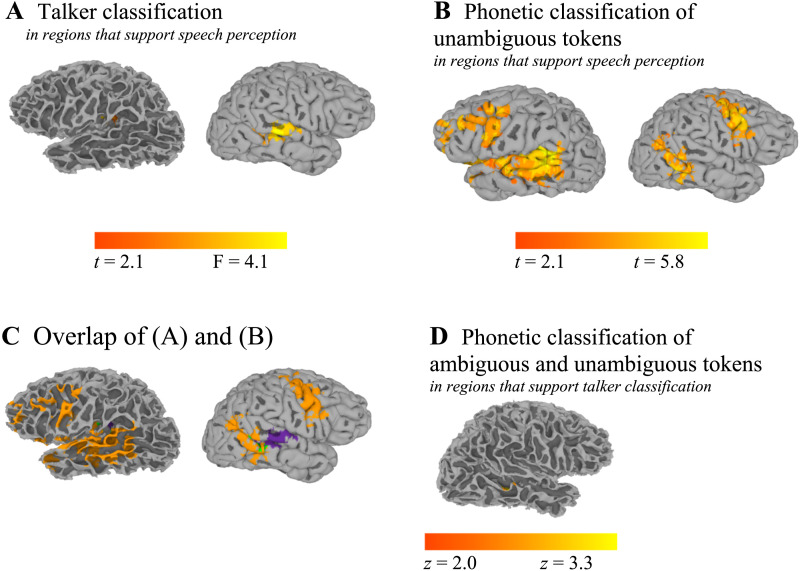
Brain regions identified using searchlight analyses. A. Set of regions sensitive to talker identity. B. Set of regions sensitive to phonetic identity. C. These two sets of regions overlapped, suggesting that some regions (left Heschl’s gyrus/insula, right superior temporal sulcus; shown in green) are sensitive both to talker information and phonetic identity. Voxels colored in purple are sensitive to talker information and voxels colored in orange are sensitive to phonetic information. D. We also conducted a searchlight analysis to identify voxels that were sensitive to phonetic identity, but for this analysis, we limited the set of candidate voxels to those identified in our talker classification analysis (panel A). By default, we show the pial surface of the brain, though for maps in which our clusters extended into sulci, we show the white matter surfaces instead. Alternate views of Figure 9D are provided in the Supporting Information.

Results of the searchlight analyses are provided in Table 4 and visualized in Figure 9. As shown in Figure 9A, we found that our classifier achieved above-chance classification of talker identity when considering only the local activation patterns in the right STG/STS (extending partly into the right MTG) as well as in early auditory cortex in the left hemisphere (left Heschl’s gyrus). A support vector machine trained to make classifications on the basis of phonetic identity performed above chance when given local activation patterns in the superior/middle temporal cortex or inferior/middle frontal cortex, whether on the left or on the right (Figure 9B). Figure 9C shows the overlap between these two classification maps; results suggest that the local patterns of activation in left primary auditory cortex or in the right STS can be used to classify trials both on the basis of talker identity (whether the listener heard the male or female talker) and on the basis of phonetic identity (whether the listener heard a clear /s/ or a clear /∫/).

**Table 4.** Results of the searchlight analyses of fMRI data (voxel-wise *p* < 0.05, cluster-level α < 0.05)

Anatomical region	Maximum intensity coordinates			Number of activated voxels	*t* value / *z* value
	*x*	*y*	*z*		
Talker classification in regions that support speech perception					
Right superior temporal gyrus / right superior temporal sulcus / right middle temporal gyrus	55	−25	8	1,005	4
Left insula / left Heschl’s gyrus	−45	−21	20	162	2.8
Phonetic classification in regions that support speech perception					
Left superior temporal gyrus / left middle temporal gyrus / left insula	−53	−39	14	3,624	4.8
Left inferior frontal gyrus / left middle frontal gyrus	−47	13	42	1,508	3.7
Right inferior frontal gyrus / right middle frontal gyrus	43	13	34	1,506	4
Right superior temporal gyrus / right superior temporal sulcus /right middle temporal gyrus	55	−49	2	1,274	3.2
Left middle frontal gyrus	−35	49	−2	370	3.6
Overlap of Talker classification and Phonetic classification maps					
Right superior temporal sulcus	53	−37	−2	67	
Left Heschl’s gyrus / left insula	−41	−25	12	62	
Phonetic classification in regions that support talker classification					
Right superior temporal sulcus	53	−37	−2	19	3.1

In a separate searchlight analysis, we attempted to classify stimuli with regard to how they were interpreted on a trial-by-trial basis (regardless of the actual acoustics). For this analysis, we only considered voxels that had been identified by the talker classification searchlight analysis (Figure 9A). As shown in Figure 9D (as well as in Figure S2), we found that voxels in the right STS could be used both for classification of phonetic identity and of talker identity; no clusters in the left Heschl’s gyrus ROI showed above-chance classification of both phonetic detail and talker. This analysis converges with the overlap analysis depicted in Figure 9C: Namely, voxels in right STS were sensitive to both talker and phonetic information. Taken together, the two sets of searchlight analyses suggest a role for the right STS in integrating talker information and phonetic identity.

### Functional connectivity analyses

#### Methods

To examine the extent to which the integration of talker information and phonetic information is achieved through interactions among multiple brain regions, we conducted a set of functional connectivity analyses. Each analysis used one of two seed regions identified through the talker classification searchlight analysis (Figure 9A)—that is, one analysis used the right STG/STS/MTG cluster as a seed, and the other used the left insula/left Heschl’s gyrus cluster as a seed region.

For each seed region, we conducted a regression analysis including several predictors. First, we included the smoothed, scaled time course of our seed region; this represents the baseline activity of the seed region. The second predictor was the convolution of the time course of the seed region with a vector of the onset times for all the phonetic categorization trials; this predictor therefore represents the activity of the seed region during the phonetic categorization trials (above and beyond its baseline activity) and is the regressor of main interest for these analyses. We also considered some regressors of no interest. First, stimulus onset times for each condition (ambiguous exposure trials, unambiguous exposure trials and each of the four continuum steps presented in the phonetic categorization task, modeled separately for each talker) were convolved with a gamma function to generated idealized hemodynamic response functions. Finally, each regression also included a third-order polynomial term (to account for scanner drift over the course of the run) as well as the six motion parameters estimated during the alignment step of preprocessing.

To identify which regions were functionally connected with each seed region during the phonetic categorization task, we conducted a one-sample *t* test on the subject-wise beta estimates for the Seed × Phonetic Categorization task regressor using the 3dttest++ command in AFNI (Cox, 1996); as in the searchlight analyses, results were masked to only include the voxels shown in Figure 8. To correct for multiple comparisons, Monte Carlo simulations were performed on the group mask using the 3dClustSim command, using average smoothness values estimated by applying the 3dFWHMx command to the residual time series data from each regression. In this way, we determined that we would need a minimum of 209 voxels for a statistically significant cluster (voxel-wise *p* < 0.05, cluster-level α < 0.05).

#### Results

**Figure 10.**
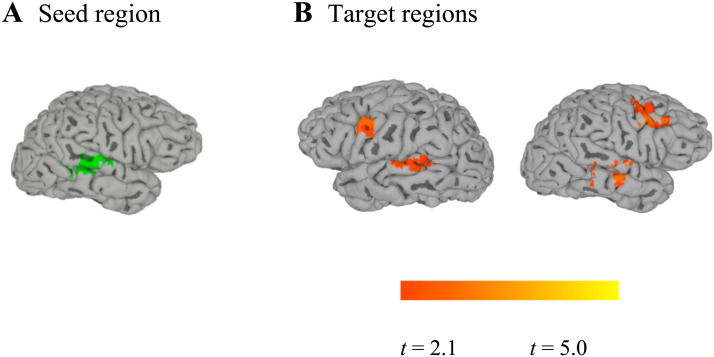
Functional connectivity analysis to investigate how the integration of phonetic information and talker information might be achieved through the coordinated activity across multiple brain regions. A. Our seed region (green) was functionally defined as the set of right hemisphere voxels that supported talker classification, as identified by our searchlight analysis. B. Target regions identified in this analysis showed increased functional connectivity with the seed region during phonetic categorization trials.

In one functional connectivity analysis, we examined which other brain regions were functionally connected with the right STG/STS seed during the phonetic categorization task. As described in Table 5 and illustrated in Figure 10, we found that on phonetic categorization trials, the activity of the right STG/STS showed significant correlations (relative to the baseline degree of functional connections) with the activity of the right MFG, the right MTG/STG, the left IFG, and the left posterior STG.

**Table 5.** Functional connectivity results (right hemisphere seed, voxel-wise *p* < 0.05, cluster-level α < 0.05)

Anatomical region	Maximum intensity coordinates			Number of activated voxels	*t* value
	*x*	*y*	*z*		
Right middle frontal gyrus	53	9	−34	692	2.2
Right middle temporal gyrus / right superior temporal gyrus	67	−27	−4	312	3.2
Left middle frontal gyrus / left inferior frontal gyrus	−53	9	−34	274	3
Left superior temporal gyrus / left middle temporal gyrus	−63	−13	−8	255	2.1

*Note*. The seed region was a right hemisphere cluster shown to support talker classification.

**Figure 11.**
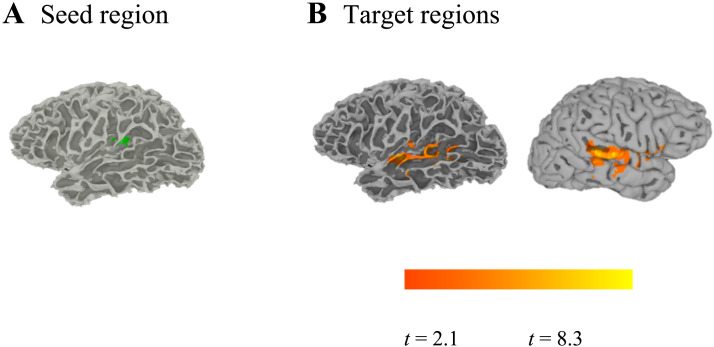
Functional connectivity analysis to investigate how the integration of phonetic information and talker information might be achieved through the coordinated activity across multiple brain regions. A. Our seed region (green) was functionally defined as the set of left hemisphere voxels that supported talker classification, as identified by our searchlight analysis. B. Target regions identified in this analysis showed increased functional connectivity with the seed region during phonetic categorization trials.

In our second functional connectivity analysis, we examined which brain regions were functionally connected with the left insula/Heschl’s gyrus seed region that had been identified by the searchlight analysis. As described in Table 6 and illustrated in Figure 11, we found that on phonetic categorization trials, the activity of the left insula/Heschl’s gyrus showed significant correlations (relative to the baseline degree of functional connections) with the activity of the left STG/MTG and the right STG/MTG/insula.

**Table 6.** Functional connectivity results (left hemisphere seed, voxel-wise *p* < 0.05, cluster-level α < 0.05)

Anatomical region	Maximum intensity coordinates			Number of activated voxels	*t* value
	*x*	*y*	*z*		
Left superior temporal gyrus / left middle temporal gyrus	−63	−11	6	943	8.3
Right middle temporal gyrus / right superior temporal gyrus / right insula	67	−27	−4	902	6.6

*Note*. Seed region was a left hemisphere cluster shown to support talker classification.

## GENERAL DISCUSSION

In this study, listeners were exposed to two talkers who produced an ambiguous fricative (“*?*”) either in place of /s/ or in place of /∫/. During exposure runs, ambiguous fricatives were encountered in disambiguating lexical frames (e.g., *epi?ode*). During test phonetic categorization runs, listeners heard these sounds in the absence of lexical context (e.g., *a?i*) and had to condition their interpretation of stimuli on acoustic details correlated with talker identity. Of interest were the patterns of functional activation elicited during the test runs. Results of searchlight analyses indicate that the patterns of activation in the right STS contain information about talker identity as well as about phonetic identity, suggesting that the right STS may serve as an interface between a left-lateralized phonetic processing system and a right-lateralized neural system for talker processing. Functional connectivity analyses examined how the activity of other regions related to the activity of the right STS when listeners made phonetic categorization decisions. Notably, we observed increased functional connectivity between the right STS and right hemisphere regions that support talker processing, as well as increased functional connections between the right STS and regions in the left hemisphere implicated in phonetic processing.

### Talker-Specific Phonetic Processing

Strikingly, the behavioral data elicited by our fMRI experiment (Experiment 2) did not comport with the results from our behavioral pilot (Experiment 1). In Experiment 1, listeners’ performance during the phonetic categorization task depended on the contexts in which they had previously encountered the speech sounds (i.e., whether lexical knowledge biased them to interpret ambiguous sounds from that talker as /s/ or /∫/) but did not depend strongly on talker’s gender (i.e., female or male). By contrast, listeners in Experiment 2 were not influenced by the lexically biasing information presenting during exposure runs but were strongly influenced by the talker’s gender.

We believe that the discrepancy in the behavioral results is most likely attributable to the insert headphones that were used in the scanner. In a follow-up headphone evaluation, we recorded stimuli directly from the insert headphones and conducted an acoustic analysis on the recordings. We found that frequencies above the 5000–6000 Hz range were almost completely attenuated by the insert headphones. Our acoustic analysis also indicated that the spectral means in our stimuli—a key acoustic property for distinguishing between /s/ and /∫/—were altered by the insert headphones, and the attenuation affect was not equal for the two talkers. From a methodological perspective, the current work reveals that researchers must characterize the frequency response profile of headphones they are using (particularly for MRI studies) and evaluate the impact this will have on stimulus presentation. If potential attenuation effects cannot be mitigated, it becomes critical to ensure during piloting that the behavioral effects of interest can still be elicited when, for example, high frequencies are attenuated. To do so, researchers might construct an auditory filter based on the frequency response profile of the MRI headphones, apply the filter to their auditory stimuli, and then conduct behavioral pilot tests to understand how the in-scanner headphones might influence the behavioral effect of interest. Researchers might also consider other (e.g., fiber-optic) headphone systems that can more faithfully deliver high frequencies, though some attenuation should still be expected, and appropriate pilot testing should still be conducted with other stimulus delivery setups. Researchers may also need to weigh potential attenuation of high frequencies against other important considerations (e.g., the quality of fit of the headphones, which will also influence the degree of scanner noise heard by the participant).

Listeners in Experiment 2 did show differential phonetic categorization profiles for the two talkers. However, these talker-specific beliefs were likely guided by acoustic details in the signal, rather than by lexical information specifically. Note that because the male talker’s speech was generated by shifting the pitch and formant ratios from the female talker’s speech, the acoustics of the two talkers’ productions differ systematically; for instance, the spectral center of gravity differs between the two talkers, as shown in Figure 7. Thus, when listeners conditioned phonetic identity on talker information (whether categorical representations of talker identity or whether simply acoustic patterns correlated with talker identity), they may have done so based on the specific acoustic properties of the stimuli. Of interest, prior research has shown that listeners can condition phonetic identity on the perceived gender of the talker as well as other socio-indexical properties (Johnson et al., 1999; Kleinschmidt, 2019); it is therefore an empirical question whether listeners’ beliefs in the current study were shaped by the raw acoustic information in the stimuli or by the acoustic details conditioned on gender (or some other socio-indexical variable). More generally, future work is needed to investigate how the neural basis of accessing talker-specific generative models may differ depending on the specific source of information (lexical, socio-indexical, acoustic) that shapes a listener’s beliefs.

### The Role of the Right Superior Temporal Sulcus

We conducted a series of searchlight analyses to investigate whether any brain regions simultaneously represented information about talker and about phonetic identity. An initial analysis found that talker identity could be recovered from local patterns of activation in the right posterior temporal cortex (primarily voxels in the STG and STS extending into the MTG) as well as from the patterns of activity in left Heschl’s gyrus. To test if any of these patterns also contained information about phonetic identity, we used both a conjunction approach and a ROI analysis. For the conjunction analysis, we examined the overlap between the voxels identified in our talker classification analysis and voxels identified in a parallel phonetic classification analysis. We found overlap between these two maps in right STS as well as in left Heschl’s gyrus. For the ROI analysis, we conducted a phonetic classification analysis within the right-hemisphere cluster (STG/STS/MTG) identified by the talker classification analysis. Here as well, we found that the local pattern of activation in the right STS could be used to perform both talker classification and phonetic classification, corroborating the results of the conjunction analysis. Overall, our results clarify the contributions of right STG, STS and MTG for processing talker information and phonetic information. In particular, they suggest that the right STG is a key region for representing talker information, that the right MTG is a key region for representing phonetic information, and that the right STS represents both sources of information. Thus, the present work indicates that the right STS may serve as an integration site for talker information and phonetic information, allowing listeners to condition phonetic identity on talker information.

Our findings support and extend results from a previous MVPA study by Formisano et al. (2008), who also found that the right STS was important for decoding both phonetic identity and talker identity. However, we differentiate our study from theirs in four key ways. While Formisano et al. presented listeners with three Dutch vowels (/a/, /i/, and /u/) produced in isolation by three talkers, we presented our speech sounds (/s/ and /∫/) in lexical contexts, thereby increasing the ecological validity of the results. Secondly, we included phonetically ambiguous stimuli in our study, whereas Formisano et al. used only unambiguous stimuli; our goal in including phonetically ambiguous stimuli was to encourage reliance on talker information, which could be used to guide the interpretation of the phonetically ambiguous stimuli. Thirdly, the present work is distinguished from the previous study by the use of a searchlight-based approach, which allows us to make stronger claims about the spatial specificity of our results. This is because a searchlight analysis tests whether above-chance classification can be achieved on the basis of the local pattern of activation. By contrast, Formisano et al. considered a large set of voxels (bilateral auditory cortex) and used RFE to identify the most informative voxels. However, voxels that are identified through this approach must be considered in the context of the full set of voxels that supported classification (Hebart & Baker, 2018). Thus, even though their analyses implicated voxels in the right STS as representing both talker identity and phonetic identity, it is possible that a classifier given only those voxels would not be able to perform above chance. The convergence between our results and those from Formisano et al. is encouraging, given differences in materials and analysis approach. Finally, we designed the current study to investigate how listeners access their beliefs of how different talkers produce their speech sounds as those beliefs were updated through perceptual learning; that is, we wanted our listeners to learn the phonetic signature of each talker during the experiment. However, as discussed above, listeners in Experiment 2 did not show the expected learning effects behaviorally, a result we suspect was driven by the particular auditory equipment used in our study. Thus, in both the present work and the work by Formisano et al., listeners’ phonetic processing likely reflects their prior assumptions of how the two talkers would produce their speech sounds rather than being driven by recently updated generative models.

While the present results suggest that the right STS plays an important role in allowing listeners to integrate phonetic information and talker information, future work will be needed to examine the generalizability of these results. For instance, previous work has shown that phonetic features such as voicing, manner, and place of articulation are distributed across the superior temporal lobe (Arsenault & Buchsbaum, 2015; Mesgarani et al., 2014), so it will be important to examine whether the right STS plays a similar role in conditioning phonetic identity on talker information for speech sound distinctions that rely on other featural differences. Additionally, it is unclear whether the right STS is *necessary* for integrating phonetic detail and talker information. Notably, our conjunction analysis suggested that phonetic information and talker information are also simultaneously represented in left Heschl’s gyrus. Thus, even when listeners are unable to recruit the right STS (whether as a result of brain damage or due to the application of focal brain stimulation), listeners might still be able to adapt to talker-specific phonetic variation if they are able to recruit left Heschl’s gyrus. More generally, an important future direction will be to delineate the differential contributions of the right STS and left Heschl’s gyrus for contacting talker-specific generative models of how acoustics map onto speech sounds.

### A Multi-System Approach to Talker-Specific Phonetic Processing

In addition to testing whether phonetic information and talker information are simultaneously encoded in the right hemisphere, we also tested the hypothesis that the process of conditioning phonetic identity on talker information is achieved through the coordinated activity of multiple brain regions. Such an investigation was motivated in part by previous work showing an increase in the functional connectivity of the left and right temporal cortices during speech processing (von Kriegstein et al., 2010). We found that on phonetic categorization trials, there was an increase in the connectivity of our right STG/STS seed region (defined functionally from our talker classification searchlight analysis) and several target regions: (1) the right MFG, a region that has been implicated for challenging vocal identity processing tasks (e.g., when listeners must hold vocal auditory objects in working memory; Stevens, 2004); (2) a slightly anterior portion of the right MTG, which is notable since anterior parts of the right temporal cortex have been linked to explicit vocal identification (see Luthra, 2021, for review), and (3) two left hemisphere regions thought to support phonetic processing: the left IFG (Lee et al., 2012; Myers, 2007; Myers et al., 2009; Rogers & Davis, 2017; Xie & Myers, 2018) and the left posterior STG (Desai et al., 2008; Liebenthal et al., 2010; Luthra et al., 2019; Mesgarani et al., 2014; Myers, 2007; Yi et al., 2019). Overall, then, when listeners performed a phonetic categorization task, there was an increase in the functional connections between: (1) a right STS region shown to represent talker information, (2) other right hemisphere regions that support vocal identity processing, and (3) left hemisphere regions implicated in phonetic processing. Taken together, these results suggest that the right STS may serve as an interface between the neural systems for talker processing and phonetic processing and that access to talker-specific generative models may be supported through the coordinated activity of these two systems. Consistent with this latter point, we also observed increased functional connectivity during the phonetic categorization task between our left Heschl’s gyrus seed region (again, defined functionally from our talker classification searchlight analysis) and the STG/MTG bilaterally.

### Conclusions

The speech signal simultaneously conveys information about who is talking and information about what is being said, and previous work suggests a substantial degree of interdependence between talker information and linguistic information (Maguinness et al., 2018; McGettigan & Scott, 2012; Mullennix & Pisoni, 1990). Several studies have demonstrated talker-specific effects in speech perception and in recognition memory (Kapnoula & Samuel, 2019; Luthra et al., 2018; Mattys & Liss, 2008; McLennan & Luce, 2005; Nygaard et al., 1994; Palmeri et al., 1993; Theodore et al., 2015), and theoretical accounts suggest that speech perception is guided by a listener’s expectations of how a talker will produce their speech sounds (Crinnion et al., 2020; Kleinschmidt, 2019; McMurray & Jongman, 2011).

An emergent body of literature has suggested that the right hemisphere may play an important role in adapting to the idiosyncratic ways that different talkers produce their speech sounds (Luthra, 2021; Luthra et al., 2020; Myers & Mesite, 2014; Myers & Theodore, 2017). In the current work, we used searchlight analyses to show that talker information and phonetic identity are both represented in the local patterns of functional activation in the right STS. We also found evidence that the integration of talker information and phonetic information may depend on the coordinated activity of this right STS region, right hemisphere regions that support vocal identity processing, and left hemisphere regions that support phonetic processing. Though theoretical models of speech perception have suggested a relatively minor role for the right hemisphere (at least in comparison to its left counterpart), the current study suggests that the right hemisphere meaningfully contributes to speech perception, particularly when listeners must adapt to talker-specific phonetic variation.

## ACKNOWLEDGMENTS

The authors thank Rachel Theodore, Christian Brodbeck, Roeland Hancock, and Dave Kleinschmidt for helpful comments on a previous version of this manuscript. We also thank Erika Skoe and Jennifer Tufts for assistance evaluating the headphone equipment used in this study. Additional thanks are extended to members of the Language and Brain Lab and the Computational Cognitive Neuroscience of Language Lab. Sahil Luthra was supported by a National Science Foundation (NSF) Graduate Research Fellowship. We gratefully acknowledge a seed grant from the Brain Imaging Research Center at the University of Connecticut. This research was supported in part by the Basque Government through the Basque Excellence Research Centres (BERC) 2022–2025 Program, and by the Agencia Estatal de Investigación (Spain) through the Basque Center on Cognition, Brain & Language (BCBL) Severo Ochoa excellence accreditation CEX2020-001010-S to BCBL.

## FUNDING INFORMATION

Emily B. Myers, National Science Foundation (https://dx.doi.org/10.13039/100000001), Award ID: 1554810. Emily B. Myers, National Institutes of Health (https://dx.doi.org/10.13039/100000002), Award ID: R01 DC013064. James S. Magnuson, National Science Foundation (https://dx.doi.org/10.13039/100000001), Award ID: NRT 1747486. James S. Magnuson’s effort was also supported in part by the Basque Government through the BERC 2022–2025 program and by the Spanish State Research Agency (https://dx.doi.org/10.13039/501100011033) through BCBL Severo Ochoa excellence accreditation CEX2020-001010-S and Award ID: PID2020-119131GB-I000.

## AUTHOR CONTRIBUTIONS

**Sahil Luthra**: Conceptualization; Formal analysis; Funding acquisition; Investigation; Methodology; Project administration; Visualization; Writing – original draft, Writing – review & editing. **James S. Magnuson**: Conceptualization; Funding acquisition; Methodology; Project administration; Supervision; Writing – review & editing. **Emily B. Myers**: Conceptualization; Funding acquisition; Methodology; Project administration; Supervision; Writing – review & editing.

## TECHNICAL TERMS

Generative model: Beliefs (likely implicit) about how certain talkers produce their speech sounds; sensitivity to acoustic patterns that correlate with talker/phonetic identity.
Lexically guided perceptual learning: Paradigm wherein listeners use vocabulary knowledge to update their generative model of how some talker produces their speech sounds.
Functional connectivity analysis: Analysis examining how well activity in a seed region predicts activity of other regions; assesses interactions between multiple brain regions.
Searchlight analysis: Form of multivoxel pattern analysis wherein a roving pattern classifier only considers the local pattern of activation.
