# Supplementary material for: Right Posterior Temporal Cortex Supports Integration of Phonetic and Talker Information
Source: Neurobiol Lang (Camb). 2023 Mar 8;4(1):145–77. doi: 10.1162/nol_a_00091 (PMC10205075; doi:10.1162/nol_a_00091)
Supplement: Supplementary file 1 [file nol-4-1-145-s001.pdf]

Supplementary Materials

Univariate Analysis

Methods

After preprocessing, we conducted a univariate regression analysis for each participant. To construct the regressors of interest, stimulus onset times for each condition (ambiguous exposure trials, unambiguous exposure trials and each of the four continuum steps presented in the phonetic categorization task, modeled separately for each talker) were convolved with a gamma function to generated idealized hemodynamic response functions. Each regression also included a third-order polynomial term (to account for scanner drift over the course of the run) as well as the six motion parameters estimated during the alignment step of preprocessing.

Group-level analyses were performed using the *3dLME* command and tested for fixed effects of Step, Bias (sh-bias, s-bias), and Talker (female, male); random by-subject intercepts were also included in the model. Results were masked to only include voxels that were (a) imaged in all 20 participants and (b) in regions that are broadly associated with speech processing – namely, the inferior frontal, middle frontal, insular, superior temporal, transverse temporal, middle temporal, supramarginal and angular cortices bilaterally, as defined in the AFNI Talairach atlas. This group mask is visualized in Figure 3.6A. To correct for multiple comparisons, we first applied the *3dFWHMx* command, which uses a spatial autocorrelation function to estimate the smoothness of the residual (error) time series from the regression analysis. To assess the likelihood of noise-only clusters, we then used the *3dClustSim* command to perform a series of Monte Carlo simulations on our group mask using the mean estimated smoothness values. These simulations

indicated that we needed at least 116 contiguous voxels for a statistically significant cluster (with a voxel-wise *p* value of 0.05 and a cluster-wise alpha level of 0.05).

Results

Our univariate analysis examined how functional activation during the phonetic categorization task depended on Step (how /s/-like or /ʃ/-like the continuum step was), Bias (whether the talker had previously produced ambiguous fricatives in /s/-biased or /ʃ/-biased contexts), and Talker (whether the female or male talker produced the stimulus). Results are summarized in Table S1 and visualized in Figure S1; the full set of voxels we considered is shown in Figure 8 of the main text.

Figure S1A shows that several regions, notably including the bilateral superior temporal cortex, showed stronger activation when speech was presented compared to silent trials. As shown in Figure S1B, a broad set of bilateral temporal regions were sensitive to the particular continuum step participants heard, as were the left middle frontal gyrus and the inferior frontal cortex bilaterally. A planned comparison contrasted the activation for the most ambiguous continuum steps (i.e., those near the /s/-/ʃ/ phonetic category boundary) and unambiguous steps (i.e., the clear /s/ and the clear /ʃ/). We found that parts of the bilateral temporal cortex responded more strongly to the unambiguous tokens than to the ambiguous ones, as did the left middle frontal gyrus (Figure S1C). We observed sensitivity to the Bias manipulation in bilateral temporoparietal cortex as well as in the bilateral inferior / middle frontal gyri (Figure S1D). By contrast, a relatively limited set of regions was differentially sensitive to which particular talker listeners heard, as shown in Figure S1E; specifically, we observed an effect of Talker in left posterior auditory cortex, the right superior and middle temporal gyri (extending into the right parietal cortex), and right inferior

frontal cortex. Finally, we observed a significant Bias × Talker interaction in the right middle frontal gyrus.

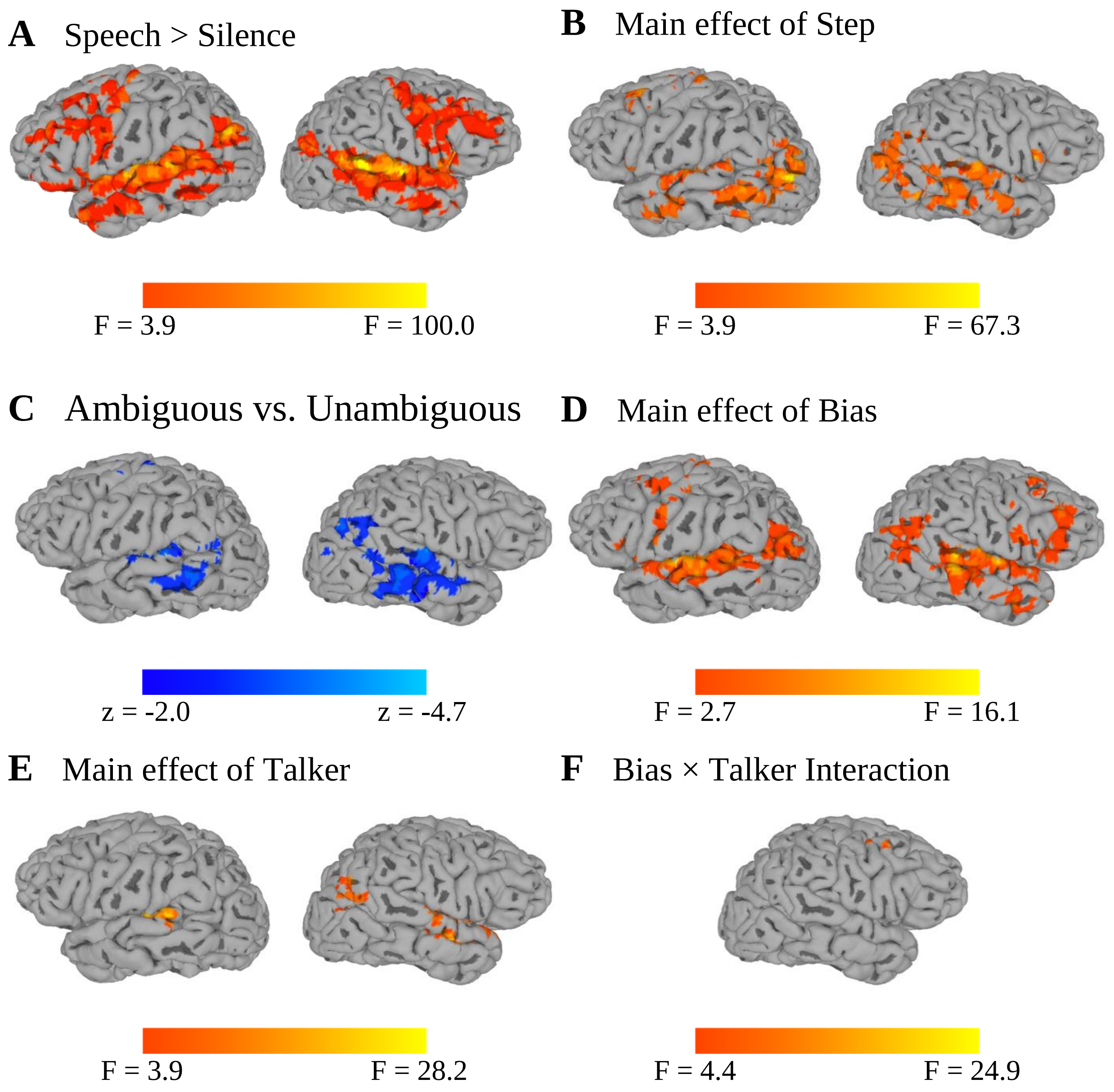

Figure S1. The univariate analysis considered activation during the phonetic categorization task and was limited to (A) left-hemisphere regions that have been implicated in language processing and the corresponding regions in the right hemisphere. Functional activation was significantly modulated by (B) which step on the *sign-shine* continuum was presented, with (C) a subset of these regions responding more strongly to unambiguous tokens than ambiguous tokens. Activation was also modulated by (D) whether the talker had previously produced ambiguous fricatives in /s/-biased or /ʃ/-biased contexts, (E) whether the male or female voice was being presented, and (F) the interaction between these two factors. Volumetric clusters were projected to a surface reconstruction using FreeSurfer (Fischl, 2012) and SUMA (Saad & Reynolds, 2012).

Anatomical region	*Maximum intensity coordinates*			*Number of activated voxels*	*F-value / z-value*
	x	y	z		
**Speech > Silence**					
1. Right superior temporal gyrus / Right insula / Right inferior frontal gyrus / Right middle frontal gyrus	57	-5	2	6713	
2. Left superior temporal gyrus / Left insula / Left inferior frontal gyrus / Left middle frontal gyrus	-45	5	0	5894	
3. Left angular gyrus	-43	-63	28	670	
4. Left anterior superior temporal gyrus / Left anterior middle temporal gyrus	-45	17	-20	539	
5. Left middle frontal gyrus	-21	25	42	474	
6. Left inferior frontal gyrus	-25	29	-8	453	
7. Right angular gyrus	45	-67	30	424	
8. Right anterior middle temporal gyrus	61	-7	-14	314	
9. Left middle temporal gyrus	-53	-39	-4	222	
**Main effect of Step**					
1. Right middle temporal gyrus / Right superior temporal gyrus	53	-57	-2	2566	11.9
2. Left middle temporal gyrus / Left superior temporal gyrus	-43	-69	16	1145	16.1
3. Left middle temporal gyrus / Left superior temporal gyrus / Left insula	-45	-17	14	936	9.5
4. Left middle frontal gyrus	-25	-9	62	323	8.9
5. Left middle frontal gyrus	-27	25	44	196	8.6
6. Left inferior frontal gyrus / Left insula	-37	23	8	175	10
7. Right inferior frontal gyrus	47	19	10	162	10.2

**Planned Comparison:** *Ambiguous vs. Unambiguous*					
1. Right supramarginal gyrus / Right superior temporal gyrus / Right middle temporal gyrus	63	-9	10	2250	-3.2
2. Left superior temporal gyrus / Left middle temporal gyrus / Left insula	-61	-11	12	846	-2.4
3. Left middle frontal gyrus	-23	11	62	209	-3
**Main Effect of Bias**					
1. Left superior temporal gyrus / Left insula	-49	-11	8	2509	67.3
2. Right superior temporal gyrus / Right insula	65	-19	6	2381	62.9
3. Right supramarginal gyrus / Right angular gyrus / Right superior temporal gyrus / Right middle temporal gyrus	45	-63	30	885	22.4
4. Left supramarginal gyrus / Left angular gyrus / Left superior temporal gyrus / Left middle temporal gyrus	-45	-71	28	583	26.2
5. Left inferior frontal gyrus / Left middle frontal gyrus	-55	7	32	285	53.7
6. Left middle frontal gyrus	-41	15	44	257	14
7. Right anterior middle temporal gyrus	49	1	-22	219	39.6
8. Left middle frontal gyrus	-17	-9	58	174	29.9
9. Right inferior frontal gyrus	57	7	26	156	35.4
10. Right anterior superior temporal gyrus	37	11	-20	154	21.9
11. Right middle frontal gyrus	27	25	44	144	23.9
**Main effect of Talker**					
1. Left superior temporal gyrus / Left Heschl’s gyrus / Left insula	-47	-7	2	582	28.2

2. Right superior temporal gyrus / Right middle temporal gyrus / Right insula	59	-9	8	439	20.6
3. Right supramarginal gyrus / Right angular gyrus / Right superior temporal gyrus / Right middle temporal gyrus	45	-53	36	275	17.4
4. Right inferior frontal gyrus / Right insula	41	9	0	146	15.8
**Bias × Talker Interaction**					
1. Right middle frontal gyrus	43	23	44	148	20.7

Table S1. Results of the univariate analysis of fMRI data (voxel-wise $p < 0.05$, cluster-level $\alpha < 0.05$).

Discussion

Univariate analyses demonstrated that bilateral temporal and frontal regions were sensitive to whether the talker had previously produced ambiguous fricatives in /s/-biased or /ʃ/-biased contexts. The clusters observed in the current study were generally comparable (albeit more widespread) than the related clusters observed by Myers and Mesite (2014), who found that differences in the response of the RIFG and the LSTG/MTG as a function of the type of biasing exposure listeners had received. However, we suggest caution in interpreting these data given that we did not observe behavioral effects of the lexically biasing exposure in our fMRI experiment.

Effects of talker identity (female or male) were observed in several regions associated with vocal identity processing (Luthra, 2021). In particular, we found sensitivity to talker gender in the posterior superior temporal lobe bilaterally, consistent with the idea that this region supports discrimination between talkers (Belin et al., 2000; Bestelmeyer, Belin, & Grosbras, 2011), as well as in the right anterior temporal lobe, which has been implicated in mapping from acoustic information to a known vocal identity (Belin & Zatorre, 2003; Luzzi et al., 2018; Van Lancker & Kreiman, 1987). We also observed an effect of talker gender in the right inferior frontal gyrus, a

region that has previously been implicated in making gender categorization on different voices (Jones, Farrall, Belin, & Pernet, 2015); notably, participants were not making gender judgments during the test blocks but had made gender categorization decisions during exposure blocks.

Finally, univariate analyses revealed that the recruitment of the right MFG was jointly influenced by which specific talker listeners were hearing (female or male) as well as whether the talker had previously produced ambiguous fricatives in /s/-biased or /ʃ/-biased contexts. The right frontal cortex is not thought to play a substantial role in phonetic processing, though it is possible that this region may be relatively more important when talker identity uniquely determines the mapping from acoustics to phonetic categories. However, we encourage caution in reading too strongly into this effect, as no effects of the specific biasing context were observed in the behavioral data. Instead, we suggest that future work should more thoroughly scrutinize the contributions of right frontal cortex to phonetic processing. To do so, it would be important to make sure the talkers' voices are perceptually matched, such that naïve listeners do not exhibit different phonetic categorization functions for the different talkers (as they did in Experiment 2 of the current study).

Searchlight Analysis

Searchlight analyses presented in the main text provide evidence that the local pattern of activation in the right superior temporal sulcus contains information about phonetic identity as well as talker. Figure S2 serves as a complement to Figure 9D, visualizing this cluster on both pial (A) and inflated (B) brain surfaces.

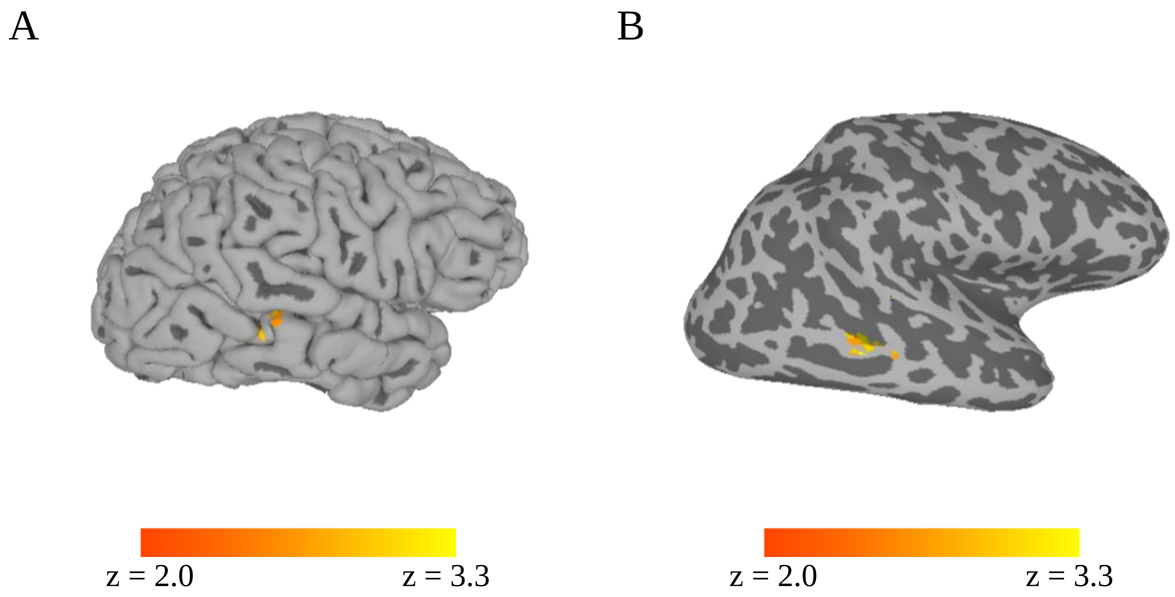

Figure S2. A set of searchlight analyses identified voxels that were sensitive to phonetic identity and talker. Here, this cluster is visualized on (A) pial and (B) inflated brain surfaces.
